# Tumor immune microenvironment-based therapies in pancreatic ductal adenocarcinoma: time to update the concept

**DOI:** 10.1186/s13046-023-02935-3

**Published:** 2024-01-02

**Authors:** Wenyu Luo, Ti Wen, Xiujuan Qu

**Affiliations:** 1 Department of Medical Oncology, The First Hospital of China Medical University, Shenyang, 110001 Liaoning China; 2 Key Laboratory of Anticancer Drugs and Biotherapy of Liaoning Province, The First Hospital of China Medical University, Shenyang, 110001 Liaoning China; 3 Clinical Cancer Research Center of Shenyang, the First Hospital of China Medical University, Shenyang, 110001 China; 4 Key Laboratory of Precision Diagnosis and Treatment of Gastrointestinal Tumors, Ministry of Education, Shenyang, 110001 Liaoning China

**Keywords:** Pancreatic ductal adenocarcinoma, Tumor microenvironment, Immunophenotyping, Integrated therapy

## Abstract

Pancreatic ductal adenocarcinoma (PDAC) is one of the most lethal solid tumors. The tumor immune microenvironment (TIME) formed by interactions among cancer cells, immune cells, cancer-associated fibroblasts (CAF), and extracellular matrix (ECM) components drives PDAC in a more immunosuppressive direction: this is a major cause of therapy resistance and poor prognosis. In recent years, research has advanced our understanding of the signaling mechanism by which TIME components interact with the tumor and the evolution of immunophenotyping. Through revolutionary technologies such as single-cell sequencing, we have gone from simply classifying PDACs as “cold” and “hot” to a more comprehensive approach of immunophenotyping that considers all the cells and matrix components. This is key to improving the clinical efficacy of PDAC treatments. In this review, we elaborate on various TIME components in PDAC, the signaling mechanisms underlying their interactions, and the latest research into PDAC immunophenotyping. A deep understanding of these network interactions will contribute to the effective combination of TIME-based therapeutic approaches, such as immune checkpoint inhibitors (ICI), adoptive cell therapy, therapies targeting myeloid cells, CAF reprogramming, and stromal normalization. By selecting the appropriate integrated therapies based on precise immunophenotyping, significant advances in the future treatment of PDAC are possible.

## Introduction

Pancreatic cancer, primarily encompassing pancreatic ductal adenocarcinoma (PDAC), is imposing an escalating global health burden. In recent years, the age-adjusted incidence of PDAC has increased with the rising of the prevalence of several key risk factors (such as smoking, obesity, and diabetes) across many regions worldwide [1]. The mortality rates of PDAC are also high as results of the insidious onset and the vast majority of patients having systemic metastasis at the time of diagnosis. In 2018, pancreatic cancer was the seventh leading cause of cancer death worldwide, which accounts for 4.5% of all cancer deaths [2], while in 2020, this proportion increased to 4.7% [3]. Due to the poor prognosis, pancreatic cancer accounts for nearly as many deaths (466,000) as the number of cases (496,000) in 2020 [3].

Currently, chemotherapy is still the main treatment for PDAC, and first-line chemotherapy includes FOLFIRINOX (oxaliplatin, irinotecan, fluorouracil, and leucovorin) or nab-paclitaxel plus gemcitabine [4, 5]. The vast majority of new chemotherapy combinations have been found to be unable to effectively improve the overall survival (OS) or progression-free survival (PFS) [6]. In the past decade, immune checkpoint inhibitors (ICI) have gradually become a key measure in treating various solid tumors, and multiple drugs targeting PD-1 (nivolumab, pembrolizumab), PD-L1 (duralumab, atezolizumab, avelumab), and CTLA-4 (tremlimumab, ipilimumab) have shown good therapeutic effects [7]. Therefore, the therapeutic effect of ICIs has also been explored in PDAC.

However, ICI standalone efficacy in PDAC is not comparable to that of other solid tumors. Studies summarized up to November 2018 found that pancreatic cancer patients treated with single-agent ICI have not been shown to elicit improvements in response rates or OS in general [8]. Besides, a phase II study found that receiving both durvalumab monotherapy and durvalumab plus tremelimumab resulted in poor prognosis and rapid disease progression, with objective response rates (ORR) of 0% (95% CI, 0.00–10.58) and 3.1% (95% CI, 0.08–16.22), respectively [9]. As no group has achieved a 10% ORR, the patients could not receive the next stage of treatments. A clear understanding of the immune microenvironment can help us understand the reasons for the failure of ICIs and discover new treatment methods. In this review, we comprehensively outline the cellular and non-cellular components in PDAC microenvironment, and highlight the latest strides in microenvironment-targeted therapeutic strategies. The recent advancements of PDAC immunophenotyping and potential treatment methods for different subtypes are summarized, with the aim of guiding precision and integrative medicine to improve patient outcomes.

## Tumor immune microenvironment of PDAC

PDAC has low mutation rate and weak immunogenicity, For example, indicators of immunotherapy effectiveness, like high-microsatellite instability (MSI) and high-tumor mutational burden (TMB), are each present in only about 1% of PDAC cases, which limits the population size of responders to immunotherapy [10, 11]. The tumor immune microenvironment (TIME) of PDAC contains tumor cells and the surrounding immune cells, fibroblasts, blood vessels, nerves and a series of active extracellular matrix components. Through intricately regulation of signaling pathways such as RAS, PI3K/AKT, NF-κB, JAK/STAT, Hippo/YAP, and WNT, tumor cells hijack the surrounding components, leading the microenvironment to develop in a direction conducive to their own growth [12]. Gradually, the immune effector cells are depleted and transformed into immunosuppressive phenotypes, or replaced by immunosuppressive cells. In addition, a large amount of matrix components is stacked in the TIME, impeding vascular perfusion. These distinctive features facilitate physical sequestration and immune evasion of PDAC, thereby impeding drug penetration and efficacy. Furthermore, the extensive heterogeneity of TIME between patients (*i.e*., significant differences in the number, proportion, distribution, and functional status of various cellular and non-cellular components) leads to significant differences in response to treatments. Given the pivotal role of the TIME during PDAC development, numerous analytical methods, including single-cell sequencing, are attempting to elucidate the specific constituents, distinctive markers and signaling interactions within the TIME. This hastens the evolution of PDAC immunophenotyping, enabling updates to treatment concepts and methodologies grounded in a deeper comprehension of the heterogeneous TIME [13].

## Preliminary immunophenotyping: simply divided into “cold” and “hot”

Due to the significant heterogeneity of TIME in PDAC, it is of utmost importance to delineate its subtypes from a clinical perspective. In fact, relevant studies on subtyping methods for PDAC are also constantly being updated, encompassing genetic, genomic, transcriptomic, morphological, proteomic, and metabolomic approaches [14]. However, none of these approaches directly encapsulate the TIME of PDAC. This further underscores the importance of developing the emerging field of immunophenotyping, where a class of patient with shared immune features can be obtained. In 2018, Wartenberg and colleagues identified three immune subtypes through next-generation sequencing and immunostaining: the “immune rich” (35%, increased T and B cells, lower mutation), the “immune exhausted” (11%, higher PD-L1 and MSI-H), and the “immune escape” (54%, decreased T and B cells, higher mutation of *CDKN2A*, *SMAD4*, and *PIK3CA*) [15]. The “immune exhausted” group can be further divided into two subgroups, one with PD-L1 expression and a high *PIK3CA* mutation rate (7%) and a microsatellite-unstable subpopulation with a high prevalence of *JAK3* mutations (4%) [15]. This study indicates that the “cold” and “hot” nature of TIME may be reflected through immunophenotyping, with different subtypes corresponding to different treatment methods. For instance, drugs targeting tumor neoantigens can be considered for the “immune rich” group, and suitable ICIs can be selected for the “immune exhausted” group, either alone or in combination with targeted drugs. For the “immune escape” group, on the basis of basic treatments, adoptive cell therapy seems to be necessary. Overall, this preliminary subtyping result provides us valuable insights: by supplementing effector T cells, enhancing endogenous T cell function, and initiating tumor specific T cell immunity, the TIME from “cold” can be transformed to “hot”, which is promising to improve the clinical treatment prospect of PDAC. In the following sections, the expanded understanding of infiltrated lymphocytes in PDAC and the therapies targeting them are summarized.

### Understanding of CD8^+^ T cell: exhaustion

**Fig. 1:**
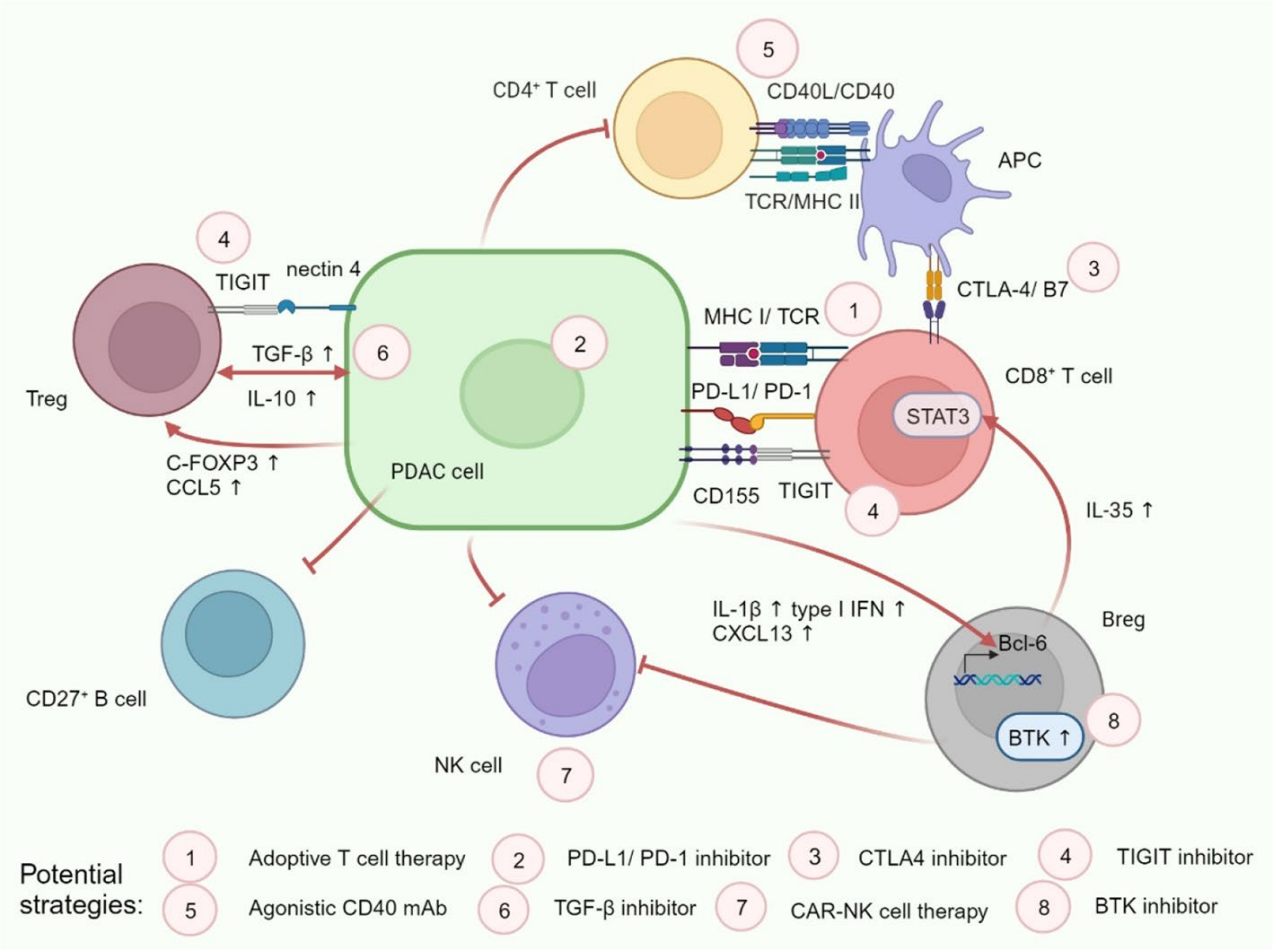
Interactions between PDAC cells and lymphocytes in TIME. Tumor cells inhibit the normal function of CD8^+^ effector T cells by upregulating the expression of multiple checkpoint ligands. Tumor cells also inhibited the activation of NK cells, CD4^+^ T cells, and B cells. By releasing a variety of immunosuppressive factors, tumor cells promote the activation of Tregs and Bregs. All these make the microenvironment present a state of immune exhaustion. Red arrows represent tumor-promoting processes. Pink circles represent possible treatment strategies. TIGIT: T cell immunoreceptor with Ig and ITIM domains; C-FOXP3: cancer Forkhead box protein 3; APC: antigen presenting cell; BTK: Bruton's tyrosine kinase; Breg: regulatory B cell

The abundant infiltration of CD8^+^ or cytotoxic T lymphocytes (CTL) is significantly correlated with improved overall survival of PDAC [16]. Nevertheless, compared to adjacent/normal tissues, the number of CD8^+^ T cells in the TIME decreases and their function is gradually exhausted. The inhibition of MHC-I exacerbates the immune escape of tumors [17, 18] (Fig. 1). Also, PDAC cells engage in intricate interactions with fibroblasts, extracellular matrix components, and multiple immunosuppressive cells to limit the activity of CD8^+^ T cells, which will be detailed in the following sections. The above reasons culminate in establishing the prevailing immune “cold” environment of PDAC. Recently, CD8^+^ tumor-infiltrating lymphocytes (TIL) were identified as displaying high expression levels of exhaustion markers such as T cell immunoreceptor with Ig and ITIM domains (*TIGIT*), *CTLA-4*, programed cell death 1 (*PDCD1*), hepatitis A virus cellular receptor 2 (*HAVCR2*), lymphocyte-activation gene 3 (*LAG3*), Layilin (*LAYN*), inducible T-cell costimulator (*ICOS*), eomesodermin (*EOMES*), and granzyme K (*GZMK*) [19, 20]. Consequently, PDAC cells adeptly evade immune killing through binding of immune checkpoint ligand receptor-ligand pairs, such as PD-1/PD-L1 or CTLA-4/B7. The CD155/TIGIT axis is also highly expressed and is key to maintaining immune evasion in PDAC cells [21]. Therefore, developing drugs targeting novel immune checkpoints like TIGIT is an innovative direction for better salvaging the depleted CD8 + T cell population together with PD-1 blockade [22, 23].

In addition, Zheng and colleagues found that most neoantigen-reactive CD8^+^ T cells presented exhausted states with significant *CXCL13* and *GZMA* co-expression compared with non-neoantigen-reactive bystander cells [24]. Schalck and colleagues found 7 CD8^+^ TIL states (CD8-GZMK, CD8-CXCR6/IL7R, CD8-ZNF683, CD8-GZMB/PRF1, CD8-CXCL13, CD8-CCR7/IL7R, and CD8-MX1) in TIME [25]. Among them, CD8-GZMK cells express EOMES and are classified as transitional “pre-dysfunctional” groups, while CD8-CXCL13 cells can maintain their proliferative potential in the early stage of dysfunction. The dysfunctional manifestations of these two populations may be the result of its repeated fighting against tumors, and thus harnessing these TIL population for therapeutic applications is valuable [25]. It is possible that different cellular states of CTLs are dynamically regulated, requiring elucidation by means such as single-cell sequencing. Building upon the concept of replenishing their numbers, tapping into the plasticity of these CTLs to reactivate exhausted subsets for improved elimination of PDAC presents an urgent challenge.

#### Combination therapy with immune checkpoint inhibitors

Enhancing patient response to ICIs is a pressing issue. Although PDAC is generally TMB-low and MSI-low, studies have shown that patients with specific genetic phenotypes may have a higher response to ICIs [26]. For instance, a non-covalent small molecule inhibitor MRTX1133 of KRAS^G12D^ has been recently found to increase CD8^+^ effector T cells, reduce myeloid cell infiltration, and reprogram fibroblasts [27]. MRTX1133 has the potential to synergistically reverse early PDAC growth with ICIs. In addition, a study enrolled 12 patients with metastatic pancreatic or biliary cancer with homologous recombination deficiency and administered ipilimumab/nivolumab. The ORR was 42% and the disease control rate (DCR) was 58%, and TILs and chemokines associated with a T cell-inflamed phenotype (CCL4, CXCL9, and CXCL10) were higher in responders than in non-responders [28]. This also suggests the possibility of applying this therapy to the “immune exhausted” populations. Two recent studies have explored the benefit of polyadenosine-diphosphate-ribose polymerase (PARP) inhibitors in patients with BRCA-mutated advanced pancreatic cancer. One of the studies found that the 6-month PFS rate of patients receiving niraparib (a PARP inhibitor) plus ipilimumab reached 59.6%, while that of patients in a niraparib plus nivolumab group was 20.6% [29]. This suggests a benefit of niraparib plus ipilimumab maintenance therapy in patients with advanced PDAC, and this benefit extends to patients without known DNA damage repair variants [29]. This study also implicates CTLA-4 as a more relevant checkpoint molecule than PD-1 or PD-L1, and CTLA-4 antibodies in combination with PARP inhibitors may induce heightened clinical efficacy. However, in another study, talazoparib combined with avelumab for the treatment of solid tumors including PDAC did not show significant improvement in efficacy [30]. The differential responses of diverse tumor lineages to PARP inhibitors underscore the need for further research into their efficacy and resistance mechanisms within the context of PDAC.

The standard regimen for combining ICIs with chemotherapy and radiotherapy is constantly under investigation. In 2020, the combination of nivolumab, nab-paclitaxel, and gemcitabine was evaluated in a phase I trial, and the median PFS and median OS were 5.5 and 9.9 months, respectively, with no apparent improvement in efficacy over chemotherapy alone [31]. Recently, the CCTG PA.7 study enrolled 180 PDAC patients and compared the efficacy of gemcitabine and nab-paclitaxel with or without durvalumab and tremelimumab. Unfortunately, the results of this phase II trial were similarly negative as combination immunotherapy did not improve survival [32]. It should be noted that chemotherapy itself has an impact on the TIME. A recent study found that patients receiving neoadjuvant chemotherapy have increased CD36 expression on the surface of tumor cells and CD8^+^ T cells, which is associated with reduced survival rate. Therefore, targeted CD36 combined with chemotherapy is expected to improve immunogenicity and enhance efficacy [33].

Encouragingly, the recent results of radiotherapy combined with ICIs have given us hope. A phase II study enrolled microsatellite stable PDAC patients and explored the effect of radiotherapy on ipilimumab plus nivolumab treatment. DCR of PDAC patients was 20% (5 of 25; 95% CI, 7–41%), and reached 29% (5 of 17; 95% CI, 10–56%) after receiving radiotherapy [34]. Meanwhile, these patients with disease control had higher NK cell numbers [34]. This study may prove to be a blessing for those “immune rich” PDAC patients characterized by low TMB. Stereotactic body radiotherapy (SBRT) appears to be an effective approach, which was evaluated in a phase II trial. It was found that SBRT plus pembrolizumab and trametinib might be a novel option for locally recurrent PDAC after surgery with a median OS of 24.9 months (95% CI, 23.3–26.5), compared with 22.4 months (95% CI, 21.2–23.6) for SBRT plus gemcitabine (hazard ratio, 0.60; 95% CI, 0.44–0.82; *p* = 0·0012) [35]. Subsequently, a study evaluated the clinical efficacy of nivolumab with or without ipilimumab in combination with SBRT for refractory metastatic pancreatic cancer. The primary endpoint of this study was clinical benefit rate, defined as the percentage of patients with complete or partial response or stable disease. The SBRT/nivolumab/ipilimumab arm demonstrated clinically meaningful anti-tumor activity and a favorable safety profile, with a clinical benefit rate of 37.2% (95% CI, 24.0–52.1%) *versus* 17.1% (95% CI, 8.0–36.0%) for SBRT/nivolumab [36]. Concurrently, decreased serum levels of IL-6, IL-8, and C-reactive protein on treatment were associated with improved OS [36]. Given the positive effects of SBRT, Zhu and colleagues investigated whether increasing doses of SBRT could elicit further improvements in survival of patients with pancreatic cancer. In this phase II trial, patients received SBRT with doses ranging from 35 to 40 Gy in five fractions. Researchers found that the dose escalation of SBRT could improve PFS with pembrolizumab and trametnib (a MEK inhibitor) *versus* gemcitabine for patients with post-operative locally recurrent pancreatic cancer [37]. However, benefits of PFS did not translate into longer OS [37]. In addition, in another phase II study, no clinically meaningful efficacy was observed in PDAC patients treated with the combination of ipilimumab, nivolumab, tocilizumab (IL-6 inhibitor), and SBRT [38]. Therefore, further research is warranted to explore the synergistic effects of SBRT with immunotherapy and targeted therapy.

Recently, a study identified receptor-interacting protein kinase 2 (RIPK2) as a crucial driver for PDAC to evade T cell killing. The ablation of RIPK2 can restore the expression of tumor MHC-I and increase the sensitivity of anti-PD-1 immunotherapy [39]. Therefore, enhancing MHC-I expression can also serve as a new therapeutic direction to promote the efficacy of ICIs.

#### Adoptive T cell therapy

Adoptive T cell therapy is the infusion of mature T cell subsets into patients to achieve the goal of eliminating tumor cells. Chimeric antigen receptor-T (CAR-T) cells have shown great promise as they are engineered in vitro for the selective recognition of target antigens on the surface of tumor cells [7]. Presently, antigen target sites under investigation for CAR-T therapy of PDAC include mesothelin, prostate stem cell antigen (PSCA), carcinoembryonic antigen, mucin 1, and HER2, among others [40]. The selection of suitable antigens remains a pivotal challenge in the CAR-T strategy. In recent years, new antigen targets for PDAC, such as stage-specific embryonic antigen-4 and CEACAM7 (members of CEA protein family) have been explored [41, 42]. In another study, CD66c, CD318 and tetraspanin 8 were identified as target candidates. The CAR-T cells specific to these molecules achieved the effect of stabilizing disease and inhibiting PDAC [43].

To overcome the immunosuppressive microenvironment, an innovative approach is to further engineer CAR-T cells to enhance T cell homing, penetration and persistence, resulting in what are known as armored CAR-T cells. Lesch and colleagues found that CAR-T cells that strongly express CXCR6 (its ligand is highly expressed by human and mouse PDAC cells and tumor-infiltrating immune cells) enhance the recognition and efficacy against tumor cells [44]. Jin and colleagues developed CAR-T cells expressing a pluripotent pro-inflammatory neutrophil-activating protein (NAP) from *Helicobacter pylori*, which exhibited slower tumor growth and higher survival rate than conventional CAR-T therapy in solid tumors including PDAC in mice, without increasing toxicity. Meanwhile, NAP facilitated dendritic cell maturation and increased infiltration of CD8^+^ T cells [45]. CAR-T cell targeting the macrophage marker F4/80 (F4.CAR-T) was developed and validated in mice, and its anti-tumor effects were mediated by the IFN-γ it produced [46]. F4.CAR-T could efficiently kill macrophages and promote expansion of endogenous CD8^+^ T cells [46]. In addition, a novel plant lectin-based CAR-T cell (H84T-CAR) was found to produce efficacy in a refractory PDAC model with aberrant glycosylation [47]. Aberrant sugar residues are a hallmark of malignant cells and associated stromal cells, while H84T-CAR efficiently disrupted these tumor cells and pancreatic stellate cells (PSC) [47]. Konduri and colleagues identified a distinct subpopulation of human CD8^+^CD161^+^ T cell by the marker CD161. CD8^+^CD161^+^ CAR-transduced T cells could kill HER2^+^ targets faster and with greater efficiency in vitro [48]. And these cells mediated enhanced in vivo antitumor efficacy in xenograft models of HER2^+^ PDAC, exhibiting higher expression of granzymes and lower expression of exhausted markers [48]. Overall, the major challenges to be addressed for CAR-T cell therapy of PDAC are the selection of neoantigens, overcoming the immunosuppressive TIME, and the issue of toxicity.

Meanwhile, researchers should also focus on the resistance of tumor itself to CAR-T cells. In one study, CAR-T cells targeting mesothelin showed dysfunction in PDAC, and this process was accompanied by a CD8^+^ T to NK-like T cell transition [49]. The investigators identified SRY-related high-mobility-group box 4 and inhibitor of differentiation protein 3 as key regulators of CAR-T cell exhaustion [49]. There is also a study that found transcription factor activating enhancer binding protein 4 to be dependent on NF-κB p65 to mediate resistance to CAR-T therapy [50]. These gave rise to the intriguing possibility of reshaping CAR-T cells through gene expression regulation for enhanced efficacy. Compared with CAR-T, T-cell receptor (TCR)-T cells offer a broader spectrum of targets because they can recognize both extracellular and intracellular antigen fragments. However, TCR-T therapy is limited to only target cancer cells that present their antigens via MHC/HLA [7]. Recently, Fujiwara and colleagues identified HLA class I and class II-restricted peptides in human PDAC, which enriched our knowledge of T-cell epitopes in PDAC and hopefully contributed to the development of TCR-T therapy [51]. Leidner and colleagues reported a case of progressive metastatic pancreatic cancer. The patient was treated with a single infusion of 16.2 × 10^9^ autologous T cells that had been genetically engineered to clonally express two allogeneic HLA-C*08:02-restricted TCRs targeting mutant KRAS^G12D^ expressed by the tumors [52]. Six months after cell infusion, the overall partial response rate reached 72%; meanwhile, the engineered T cells constituted more than 2% of all circulating peripheral-blood T cells [52]. The outcomes of this case are promising, offering a feasible paradigm for the application of TCR-T therapy. In conclusion, ICIs and adoptive T cell therapy have indeed brought great hope for the use of PDAC in immunotherapy. Moving forward, it is imperative to explore the intricacies of T-cell dysregulation within the TIME, which could potentially unveil novel lines of treatment. The amalgamation of emerging targeted therapies with existing immunotherapies holds the promise of yielding improved outcomes, seeking to achieve enduring efficacy while minimizing treatment-associated challenges.

### Dual rules of regulatory T cell (Treg)

The expression of Tregs increases during PDAC progression and correlates with poor prognosis. PDAC cells could actively express cancer Forkhead box protein 3 (C-FOXP3), CCL5, IL-10, and TGF-β to recruit and transform Tregs [53, 54]. In turn, Tregs can promote PDAC development through multiple pathways [55]. Single-cell sequencing has found the expression of some characteristic markers in Tregs, including *TIGIT*, *CTLA4*, *FOXP3*, *TNFRSF18*, *LAYN*, *DUSP4*, *FANK1* and *LAIR2*. Among them, TIGIT expressed by Tregs can interact with nectins (especially nectin4) expressed by tumor cells [19, 56, 57]. TIGIT-nectin axis hampers T and NK effector function, thereby exacerbating tumor immune escape [56]. Approaches to target Tregs appear feasible. However, Zhang and colleagues obtained a surprising finding that Tregs elimination failed to relieve immunosuppression and led to accelerated PDAC development. Tregs are a key source of TGF-β. TGF-β plays a role in suppressing tumor growth due to a driving effect on myofibroblastic fibroblasts early in carcinogenesis, which can be reversed by the elimination of Tregs [58]. Meanwhile, the loss of Tregs enabled the up-regulation of *Ccl3*, *Ccl6*, and *Ccl8* in cancer cells and fibroblasts to recruit more immunosuppressive myeloid cells, an effect that could be inhibited by the blockade of CCR1 [58]. This suggests that Tregs have a “suppressive” effect on other immunosuppressive cells. At the same time, this phenomenon indirectly suggests the rationale behind the failure of combining the TGF-β receptor inhibitor galunisertib with ICIs [59]. Therefore, implementing a combination targeted therapy based on a clear understanding of the signaling regulation mechanism of PDAC seems to be a more appropriate choice in overcoming compensatory immunosuppression in later stages of PDAC.

### Other lymphocyte types: novel immune targets

The function of NK cells in the TIME is also suppressed. Similar to CD8^+^ T cells, NK cells in the TIME express multiple immune checkpoint receptors, such as *CD47*, *TIGIT*, *TNFRSF18*, and *LAG3* [19]. The exploration of targeting NK cells in PDAC is an emerging field. However, tumor cells impede NK cell homing to tumor sites, thereby preventing sustained anti-tumor immune responses. Methods and technologies that promote NK cell homing and activation, such as NK-cell-recruiting protein-conjugated antibody (to facilitate homing) and engineered nanogel that inhibited prostaglandin E2 secretion (to promote activation), were found to improve the therapeutic effect of PDAC and increase survival [60, 61]. Recently, Teng and colleagues engineered CAR-NK cells that target PSCA and express soluble (s) IL-15 (PSCA CAR_s15 NK cells). Researchers found that PSCA CAR_s15 NK cells showed tumor-suppressive effects and prolonged survival in a human metastatic pancreatic cancer model, with no signs of systemic toxicity. This provides a strong rationale to support clinical development [62]. Another recent study implied that circulating tumor cells and NK cells interact via the immune checkpoint molecule pair HLA-E:CD94-NKG2A. These tumor cells thereby evade NK cell-mediated killing and increase metastasis [63]. Blocking this checkpoint is expected to mitigate the rate of postoperative metastasis.

Although B cells in the TIME of PDAC are mainly plasma cells and memory B cells that highly express CD27, several studies have found that IL-1β and type I interferons up-regulated CXCL13, inducing more regulatory B cell (Breg) recruitment to exert immunosuppressive functions [64, 65]. Bregs could stimulate STAT3 signaling in themselves and CD8^+^ T cells through IL-35. This resulted in two outcomes: a) the transcriptional regulator *BCL-6* is up-regulated in naive B cells, which disrupts B cell differentiation into plasma cells; b) the function of CTLs is inhibited [66, 67]. A recent study found that the resistance to stimulator of interferon gene (STING) agonists in PDAC is due to its induction of IL-35^+^ B cell expansion. Systemic anti-IL-35 and STING agonist can synergistically inhibit Breg amplification and enhance the efficacy of NK cells [68]. In addition, a phase III trial explored the effect of ibrutinib, a Bruton’s tyrosine kinase inhibitor, in inhibiting the immunosuppressive B cells. However, ibrutinib/nab-paclitaxel/gemcitabine did not improve OS or PFS in patients with PDAC compared with controls [69]. This once again demonstrates the vast heterogeneity of cellular components and complexity of immune signaling in the PDAC TIME.

Collectively, the various components in the TIME interact intricately, with one cell population transitioning to another state as PDAC progresses. Leveraging innovative techniques like single-cell sequencing and multi-omics analysis, we are poised to gain a more profound understanding of the lymphocyte profile in PDAC. Consequently, we can isolate cells with greater precision and map out cell trajectories by identifying specific markers. This advance will furnish a more robust foundation for devising immunotherapies that target the various components of the TIME.

## Progress in immunophenotyping: understanding of myeloid cells

Myeloid cells in the TIME primarily comprise tumor-associated macrophages (TAM), tumor-associated neutrophils (TAN), tumor-infiltrating dendritic cells (DC), myeloid-derived suppressor cells (MDSC), etc. During the development of PDAC, these myeloid cells contribute to an increasingly immunosuppressive microenvironment through interactions with tumor cells, thereby perpetuating a detrimental cycle. Therefore, refining the immunophenotyping approach requires comprehensive consideration of the distinct expression patterns exhibited by various immune cells within the TIME. In 2019, Santiago and colleagues identified three immune phenotypes through a meta-analysis of transcriptional subtypes of PDAC. Subtype 1 (innate immunity) showed an enrichment of innate immune cells, an exclusion of activated CD4^+^ T cells, CD8^+^ T cells and B cells, and high expression of tumor-promoting factors such as TGF-β; subtype 2 (T cell dominant) exhibited enrichment of TILs; while subtype 3 (tumor dominant) showed a paucity of TILs [70]. Notably, subtypes 2 and 3 correspond closely to the “immune rich” and “immune escape” types mentioned in previous studies, respectively. Additionally, the “innate immunity” phenotype suggested the feasibility of the combination with other immune cell-targeted therapies. By focusing on the highly expressed innate immune cells within this subtype, tailored therapeutic strategies can be designed to enhance the activation of effector T cells. In the following section, the diverse myeloid cell populations present in the TIME are summarized and targeted therapeutic approaches with which to address them were explored.

### TAMs: a vicious circle promoting PDAC develpoment

**Fig. 2:**
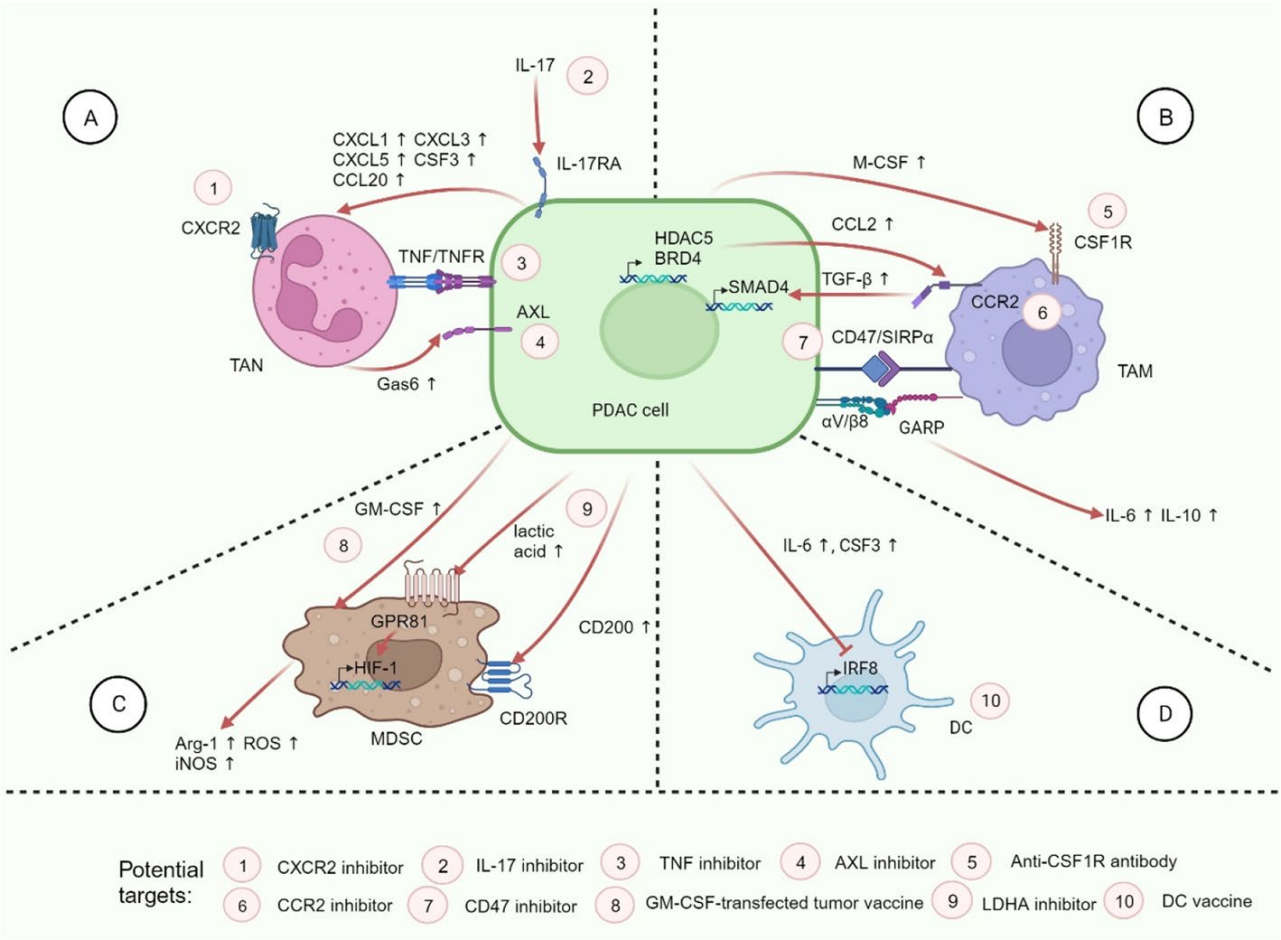
Interactions between PDAC cells and myeloid cells in TIME. **A** IL-17 receptor activation of tumor cells promotes the recruitment and activation of TANs through a variety of downstream secreted factors. TANs interact with tumor cells through TNF/TNFR, leading to the production of more chemokines. Activated TANs promote liver metastasis of PDAC by releasing Gas6 to bind to AXL on the surface of tumor cells. **B** Tumor cells promote TAM polarization toward the M2 phenotype by secreting tumor-promoting factors to activate receptors such as CSF1R and CCR2 on TAMs. The recognition of CD47/SIRPα enables tumor cells to acquire the ability of anti-phagocytosis. TAMs can also activate proliferative genes in tumor cells. **C** Tumor cells actively secrete GM-CSF and many other factors to recruit and activate MDSCs. Activated MDSCs secrete a variety of tumor-promoting factors to further induce tumor proliferation. **D** Tumor cells inhibit the activation and normal function of DCs. Red arrows represent tumor-promoting processes. Pink circles represent possible treatment strategies. TAN: tumor‐infiltrating neutrophil; TNF: tumor necrosis factor; Gas6: growth arrest specific 6; TAM: tumor-associated macrophage; GARP: Glycoprotein A repetitions predominant; MDSC: myeloid-derived suppressor cell; HIF: hypoxia-inducible factor; ROS: reactive oxygen species; Arg-1: arginase-1; iNOS: inducible nitric oxide synthase; IRF8: interferon regulatory factor-8

TAMs include macrophages generated by monocyte differentiation and tissue-resident macrophages. TAMs in PDAC can be divided into an antitumor M1 phenotype (activated by lipopolysaccharide, TNF-α and IFN-γ, expressing higher levels of IL-12, IL-23, MHC II, and inducible nitric oxide synthase) and a tumor-promoting M2 phenotype (activated by IL-4 and IL-13, expressing higher levels of IL-10 and TGF-β) [71]. During PDAC progression, tumor cells and immunosuppressive TAMs promote each other, forming a vicious circle (Fig. 2).

The mechanisms by which TAMs polarize from M1 to M2 phenotype are constantly being elucidated. It has been known that macrophage colony-stimulating factor (M-CSF)-CSF1R and CCL2-CCR2 signals induced by tumor cells stimulate TAM recruitment. Rodriguez and colleagues found that sialic acid expression is up-regulated in PDAC cells, which drives monocytes to secrete IL-10 and IL-6 and differentiate into immunosuppressive TAMs through recognition of siglec-7 and siglec-9 [72]. Additionally, PDAC cells can selectively induce DNA methylation and down-regulate oxidative phosphorylation (OXPHOS) genes in M1 TAMs through direct cell-to-cell contact. This interaction is mediated by Glycoprotein A repetitions predominant (GARP) and integrin αV/β8 and results in the suppression of the glucose metabolic state of M1 macrophages and thus their switch to the M2 phenotype [73].

M2 type TAMs have previously been shown to exhibit PDAC promoting effects through various immunosuppressive signaling pathways and effector molecules, such as: toll-like receptor (TLR)4, STAT3, TGF-β, IL-10, CCL17, CCL22, and arginase-1 (Arg-1) [71, 74]. In addition, tumor cell surfaces widely express CD47, which can bind to signal-regulatory protein α (SIRPα) on macrophages to inhibit phagocytosis. Recently, a study identified a novel histone deacetylase 5 (HDAC5)-CCL2/CCR2-TGF-β/SMAD4 axis driven by epigenetic regulation to promote tumor growth in a PDAC model. Mechanistically, HDAC5 in tumor cells inhibits *Socs3*, a negative regulator of *CCL2*, resulting in a shift from neutrophils to CCR2-expressing macrophages [75]. These macrophages express abundant TGF-β that activates SMAD4 signaling in PDAC cells and enables *KRAS*-independent tumor growth [75]. In essence, TGF-β drives SMAD4 signaling more effectively to protect tumor growth when *KRAS* is strongly inhibited. The results of this study propose a potentially effective therapeutic strategy for PDAC, namely dual inhibition of the KRAS and TGF-β/SMAD4 signaling pathways. Notably, Tu and colleagues found that PDAC cells could recruit TNF-α-secreting macrophages through the Bromodomain-containing protein 4 (BRD4)-cJUN-CCL2 axis. The abundant expression of TNF-α forced tumor cells to develop towards a poorly differentiated and aggressive basal-like phenotype [76]. This further underscores the importance of targeting the CCL2 pathway to impede PDAC development, which could be realized by inhibition of BRD4 and cJUN. Furthermore, TAMs are widely involved in the metastasis of PDAC by promoting desmoplasia, angiogenesis, lymphangiogenesis and epithelial mesenchymal transition [71].

#### Targeting TAMs: to restore normal function

Macrophage-targeted therapies are mainly categorized into three directions: a) inhibiting tumor recruiting macrophages, b) restoring the antitumor ability of macrophages, and c) reprogramming macrophages [77]. CSF1R and CCR2 are two important targets for TAM recruitment. One study evaluated the efficacy of AMG 820, an anti-CSF1R monoclonal antibody, for solid tumors including PDAC and found that AMG 820 (1100 mg) plus pembrolizumab (200 mg) combination dose had an acceptable safety profile [78]. In another phase 1b/2 study, Johnson and colleagues only observed limited clinical benefit with the CSF1R inhibitor ARRY-382 plus pembrolizumab, but it was well tolerated [79]. In the case of CCR2, its inhibitors CCX872 and PF-04136309 showed better therapeutic effect and clinical prognosis in combination with FOLFIRINOX [80, 81]. Interestingly, Wang and colleagues recently found that CCR2 and CCR5 can be induced in PDAC after treatment with αPD-1. Therefore, the researchers focused on BMS-687681, a dual antagonist of CCR2/5, in combination with αPD-1 and radiotherapy as a novel therapeutic strategy and found that it enhanced intra-tumoral effector and memory T cell infiltration but inhibited Treg, M2 TAM, and MDSC infiltration [82]. Moreover, Zhang and colleagues engineered CCR2-targeting ultrasmall copper nanoparticles as nano-vehicles that could be able to enhance detection and drug delivery of PDAC, showing great translational potential [83].

Restoration of the normal function of macrophages has been mainly achieved by targeting CD40 and CD47/SIRPα signal. CD40, as an important costimulatory molecule on the surface of antigen-presenting cells, can be activated to improve the effectiveness of immunotherapy. Recently, Selicrelumab (an agonistic CD40 monoclonal antibody) is being evaluated as a neoadjuvant therapy that induces T-cell activity and proliferation in the TIME, more mature DCs, decreases the number of M2 TAMs, and reduces tumor fibrosis [84]. Sotigalimab (APX005M) was evaluated in a phase 1b trial and was found to be tolerable and showed clinical activity in metastatic PDAC in combination with chemotherapy, with or without nivolumab [85]. However, in a subsequent phase II study, the sotigalimab/nivolumab/chemotherapy arm did not show a meaningful improvement in 1-year OS rate (41.3%, *P* = 0.223, *n* = 35) compared to nivolumab/chemotherapy (57.7%, *P* = 0.006, *n* = 34) and sotigalimab/chemotherapy (48.1%, *P* = 0.062, *n* = 36) [86]. This study suggests that only a subset of patients may fully benefit from these regimens, emphasizing the need for identifying biomarkers to select suitable candidates for sotigalimab treatment. A recent study also confirmed that the addition of CD40 antibody to irreversible electroporation (IRE) could improve DC activation and neoantigen recognition in a mouse model, while generating a strong systemic anti-tumor T cell response [87]. This implies the feasibility of a clinical trial combining IRE with local delivery of CD40 antibody. The combination of a CD40 agonist and the FMS-like tyrosine kinase 3 ligand has also been found to enhance tumor immunity and responsiveness to radiotherapy by mobilizing conventional DCs [88, 89]. In addition, Jiang and colleagues found that cobimetinib (a MEK inhibitor) and mefloquine (autophagy inhibitor) could activate the STING/type I interferon pathway in PDAC cells, thereby polarizing TAMs toward the M1 phenotype [90]. This switch is further enhanced by CD40 agonists, and this triple therapy enhances antitumor immunity [90]. Novel approaches that utilize oncolytic herpes simplex virus-1 or nanotechnology to load CD40 agonists have also all demonstrated good effects in mouse models [91, 92].

The blockade of CD47 can increase the efficiency of macrophages in clearing PDAC cells. Studies have identified strategies to utilize metabolic or epigenetic pathways for activating anti-tumor macrophages. The TLR9 agonist CpG oligodeoxynucleotide can cause changes in the central carbon metabolism of macrophages to overcome inhibitory CD47 on PDAC cells [93]. In addition, ASPEN-01 study evaluated the effect of evorpacept (a high-affinity CD47 blocking protein) in patients with solid tumors, including PDAC. Evorpacept in combination with pembrolizumab or trastuzumab demonstrated safety and preliminary anti-tumor activity [94]. Furthermore, a study found that SIRPα is also a major regulator of tumor resistance to radiotherapy. Upon radiotherapy-mediated activation, SIRPα-deficient macrophages in TIME acquire powerful proinflammatory features and conduct antigen presentation that confer a tumoricidal environment highly infiltrated by tumor-specific CTLs, NK cells and inflammatory TANs [95]. This suggests that targeting the CD47-SIRPα signaling may be combined with radiotherapy for better outcomes.

Reprogramming TAMs, that is, targeting related molecules and signals, converts TAMs from M2 to M1 phenotype. PI3K-γ is an important factor in inducing M2 TAM polarization. The PI3K-γ inhibitor IPI-549 exhibited reduced suppressive myeloid cells and retarded tumor growth in a mouse PDAC model [96]. In another study, Li and colleagues developed dual blockade with a PI3K-γ inhibitor and CSF1R-siRNA, which significantly reduced M2 TAMs and increased M1 TAMs compared with single pathway blockade [97]. In addition, approaches targeting IL-3, IL-4, TGF-β, Notch pathway, IL-27, TLR4, STAT3, and STING are all promising candidates for application in the remodeling of TAMs [98, 99].

### TANs: discovery of new subtypes

TANs in PDAC abundantly express *CXCR1*, *CXCR2*, *FCGR3B*, and *S100A8* genes, with CXCR2 being considered the key receptor for recruiting TANs [19]. Zhang and colleagues found that IL-17 recognized IL-17RA on the surface of tumor cells. Upon tumor cell activation, a large number of CXCL5, CXCL3, CSF3, CCL20, and CXCL1 are expressed to recruit neutrophils [100]. In general, TANs have a promoting effect on the development of PDAC, including remodeling the extracellular matrix, promoting tumor cell invasion and metastasis, induction of angiogenesis and lymphangiogenesis, and establishing an immunosuppressive microenvironment [101]. Recent studies identified tumor necrosis factor (TNF) derived from TANs as central immune regulators that contribute to feed-forward CXCL1 overproduction by tumor cells and fibroblasts via transmembrane TNF-TNFR2 interactions. CXCL1 subsequently mediated the expansion of MDSCs and the dysfunction of CD8^+^ T cells [102]. Studies of the role of neutrophils on PDAC murine liver metastasis model have also yielded some interesting results. Single-cell RNA sequencing of PDAC liver metastases demonstrated that metastatic PDAC tumors got immune invasion by accumulating a subpopulation of immunosuppressive *P2RX1*^*−*^ (an ATP-gated ion channel) neutrophils [103]. In vivo, chemotherapy induces initial infiltration of pro-inflammatory macrophages into the liver and consequent activation of effector T cells, resulting in temporary suppression for PDAC metastasis. However, after cessation of treatment, neutrophils are recruited to the metastatic liver via CXCL1 and CXCL2 secreted by metastatic tumor cells [104]. These TANs express growth arrest specific 6 (Gas6), which leads to AXL receptor activation on tumor cells, enabling their regeneration [104].

Recently, research into TAN subtypes has emerged as a new focal point. IRE has been shown to induce substantial infiltration of neutrophils into the pancreatic TIME. These TANs are then polarized by TGF-β into a tumor-promoting phenotype [105]. This phenomenon contributes significantly to tumor recurrence following IRE treatment. However, TGF-β inhibition can promote the remodeling of TAN into an anti-tumor phenotype, which enhances the efficacy of combined IRE + αPD1 treatment and induces long-term anti-tumor memory [105]. Wang and colleagues performed a deeper exploration of TAN heterogeneity in PDAC patients. They found a terminally differentiated pro-tumor subpopulation (TAN-1) associated with poor prognosis, an inflammatory subpopulation (TAN-2), a population of transitional stage that have just migrated to tumor microenvironment (TAN-3) and a subpopulation preferentially expressing interferon-stimulated genes (TAN-4) [106]. Hypoxia-induced hypoxia-inducible factor (HIF)1α activation and endoplasmic reticulum stress are pivotal triggers for the polarization of neutrophils toward the TAN-1 phenotype. The protumor TAN-1 subpopulation exhibits highly activated glycolytic activity, and the multiple energetic metabolites it produces can be taken up by cancer cells as an alternative to the tricarboxylic acid cycle substrates to fuel energy production and tumor growth [106]. This study provides evidence for the continuum of neutrophil transition states in the PDAC microenvironment and suggests a potential approach to target the glycolysis of TANs for tumor suppression.

#### Targeting TANs: CXCR2

At present, strategies for targeting TANs mainly focus on CXCR2, which is predominantly expressed on TANs and other myeloid cells. CXCR2 inhibition was shown to delay the progression of PDAC and enhance the sensitivity of immunotherapy [107, 108]. Gulhati and colleagues demonstrated that SX-682 (a clinical stage CXCR1/2 inhibitor) potentiated the efficacy of 41BB agonist and LAG3 antagonist, and this triple combination resulted in an almost complete depletion of CXCR2^+^ myeloid cells within murine PDAC, accompanied by a significant increase in CD8^+^ and CD4^+^ T cell infiltration [109]. It is noteworthy that a study found that targeting CXCR2^+^ TANs, or CCR2^+^ TAMs alone, resulted in a compensatory increase in alternative myeloid subsets [110]. Therefore, the use of CXCR2 inhibitors in combination with CCR2 inhibitors is recommended to reduce the overall suppressive myeloid cell count, thus fostering a more robust anti-tumor immune response in PDAC. In addition, lorlatinib, as an FDA approved third-generation ATP-competitive small-molecule tyrosine kinase inhibitor, can attenuate PDAC progression by inhibiting the development and mobilization of TANs [111]. Tintelnot and colleagues discovered that microbiota-derived indole-3-acetic acid is oxidized by myeloperoxidase expressed by neutrophils, which leads to accumulation of reactive oxygen species (ROS) and down-regulation of autophagy in PDAC cells, impairing metabolic fitness and proliferation of PDAC [112]. This study establishes a link between diet, the microbiota, and neutrophil function, and dietary modulation may serve as a harmless approach to reshaping the TIME. Notably, we need to consider the heterogeneity in the process of targeting TANs, with an aim to eliminate or reprogram the immunosuppressive TAN subgroup.

### DCs: developing vaccines

DCs are relatively rare in the TIME of PDAC, which may be one of the reasons for the ineffectiveness of immunotherapy. Single cell sequencing has identified type I conventional dendritic cells (cDC1) (expressing *CLEC9A* and *IRF8*) and cDC2s (expressing immune checkpoint ligand *SIRPA*). In addition, two populations of plasmacytoid DCs (*IRF8*, *GZMB*), two populations of Langerhans-like DCs (*CD207*, *CD1A*) and two unique populations of activated DCs (*LAMP3*, *CCL22*) were also found [19]. This is also suggestive of the functional complexity imparted by DC heterogeneity. cDC1s are necessary for tumor antigen trafficking to draining lymph nodes, antigen cross-presentation, and CD8^+^ T cell activation [88]. A study found that cDC1 dysfunction could occur even in the early stages of pancreatic intraepithelial neoplasia and that tumor formation was accompanied by elevated IL-6 evoked cDC1 apoptosis in mice [88]. While during PDAC progression, tumor-produced CSF3 down-regulated interferon regulatory factor-8 (IRF8) in cDC progenitors, leading to slower cDC1 development and reduced numbers [113]. The absence of cDCs leads to dysfunctional immunosurveillance, during which Th17 cells and their secreted IL-17 increase, further accelerating PDAC progression [89]. Given the significant role of DCs, DC vaccines have been proposed as a treatment option for PDAC, which enhance tumor antigen presentation and T cell responses by replacing damaged DCs in the TIME, increasing the expression of costimulatory molecules and proinflammatory cytokines. DC vaccines were found to improve the infiltration of CD8^+^ T cells and increase the efficacy of CD40 agonists in murine PDAC models [114]. Allogeneic lysate-DC vaccines have also preliminarily demonstrated feasibility in phase I trials [115].

Under specific circumstances, DCs might exhibit immunosuppressive properties. For instance, activated DC1s could elevate the expression of almost all immune checkpoint ligands including *PVR*. Actually, myeloid cells such as DCs, macrophages, and neutrophils are important sources of immune checkpoint ligands for human PDAC [19]. In addition, Barilla and colleagues identified a CD11b^+^CD103^−^ DC subset in murine PDAC that could induce IL-10^+^IL-17^+^ FoxP3^neg^ regulatory CD4^+^ T cell tolerance through high expression of IL-23 and TGF-β [116]. Therefore, it becomes imperative to examine the immunosuppressive impact of specific DC subpopulations.

### MDSC: enhancing immunosuppression

MDSCs are a heterogeneous cell population consisting of immature macrophages, granulocytes, and dendritic cells that exert immunosuppressive functions in the TIME. MDSCs are universally stimulated to differentiate by tumor-derived GM-CSF and recruited to the microenvironment. By releasing inhibitory factors such as Arg-1 (dependent on the STAT3 signal), ROS, and inducible nitric oxide synthase (iNOS), MDSCs could up-regulate expression of PD-L1, promote the development of M2 TAMs and Tregs, and induce exhaustion of CD8^+^ T cells and NK cells [71, 117]. A study found that CD200 (a regulator of myeloid cell activity) expressed by tumor cells and SMA^+^ stromal cells was up-regulated in the microenvironment, which stimulated CD200R-expressing MDSC expansion, especially that of CD15^+^ MDSCs [118]. Thus, approaches targeting CD200 hold promise to enhance the efficacy of immunotherapy by limiting MDSC expansion. S100A8/S100A9 heterodimer is one of the important markers of MDSC, and its high expression is positively correlated with the occurrence of PDAC cachexia [119]. Studies have found that CD74 is an upstream signal of S100A8/S100A9, which promotes its secretion through the NF-ĸB pathway. S100A8/A9 can interact with PDAC cells and CAFs, leading to the expression of various pro-inflammatory cytokines (such as IL-6, IL-8, and TNF-α), while knockout of CD74 delay the growth of tumor [120, 121]. In addition, Yang and colleagues found that the mechanism of radio-resistance in PDAC was associated with MDSCs, with lactate and HIF-1α as key mediators. The enhanced Warburg effect of radiotherapy may lead to a sustained increase in lactate secretion, which induces the activation of MDSCs via G protein-coupled receptor 81/mTOR/HIF-1α/STAT3 pathway, making the microenvironment more immunosuppressive [122]. Therefore, inhibiting the process of lactate metabolism can reverse the Warburg effect, thus regulating tumor cell metabolism and reprogramming MDSCs. This strategy holds the potential to mitigate radiation resistance in PDAC.

Inhibiting GM-CSF can significantly reduce the recruitment of immunosuppressive myeloid cells like MDSCs. GM-CSF-transfected pancreatic tumor vaccine (GVAX) is widely used in PDAC research at present. GVAX has been evaluated in multiple trials in combination with different therapies such as cyclophosphamide, CRS-207 (live, attenuated *Listeria monocytogenes* expressing mesothelin), ivolumab, and ipilimumab, however, we failed to find a clear improvement in OS [123, 124]. A study found that after receiving GVAX and CRS-207 treatment, patients with a higher abundance of CD8^+^CD45RO^−^CCR7^−^CD57^+^ cells and a lower abundance of CD8^+^CD14^+^CD33^+^CD85j^+^ cells had improved OS [125]. This suggests the potential for identifying suitable vaccination populations and predicting patient prognosis through biomarker screening. Subsequent studies unveiled that the OS of patients after GVAX vaccination was negatively correlated with TANs and positively correlated with tertiary lymphoid aggregates and the number of CD137^+^ T cells [126, 127]. Therefore, the combination of GVAX with TAN modulators and T cell activators might be able to exert greater therapeutic effect compared with single GVAX [126]. In conclusion, both adaptive and innate immunity collectively regulate the tumor immune response in the TIME. Thus, in-depth studies on myeloid cells contribute to a comprehensive understanding of PDAC immune regulation, which, in turn, will facilitate the development of therapies for the re-education or reprogramming of immune cells in subsequent stages.

## More complete immunophenotyping: enrolling the matrix

**Fig. 3:**
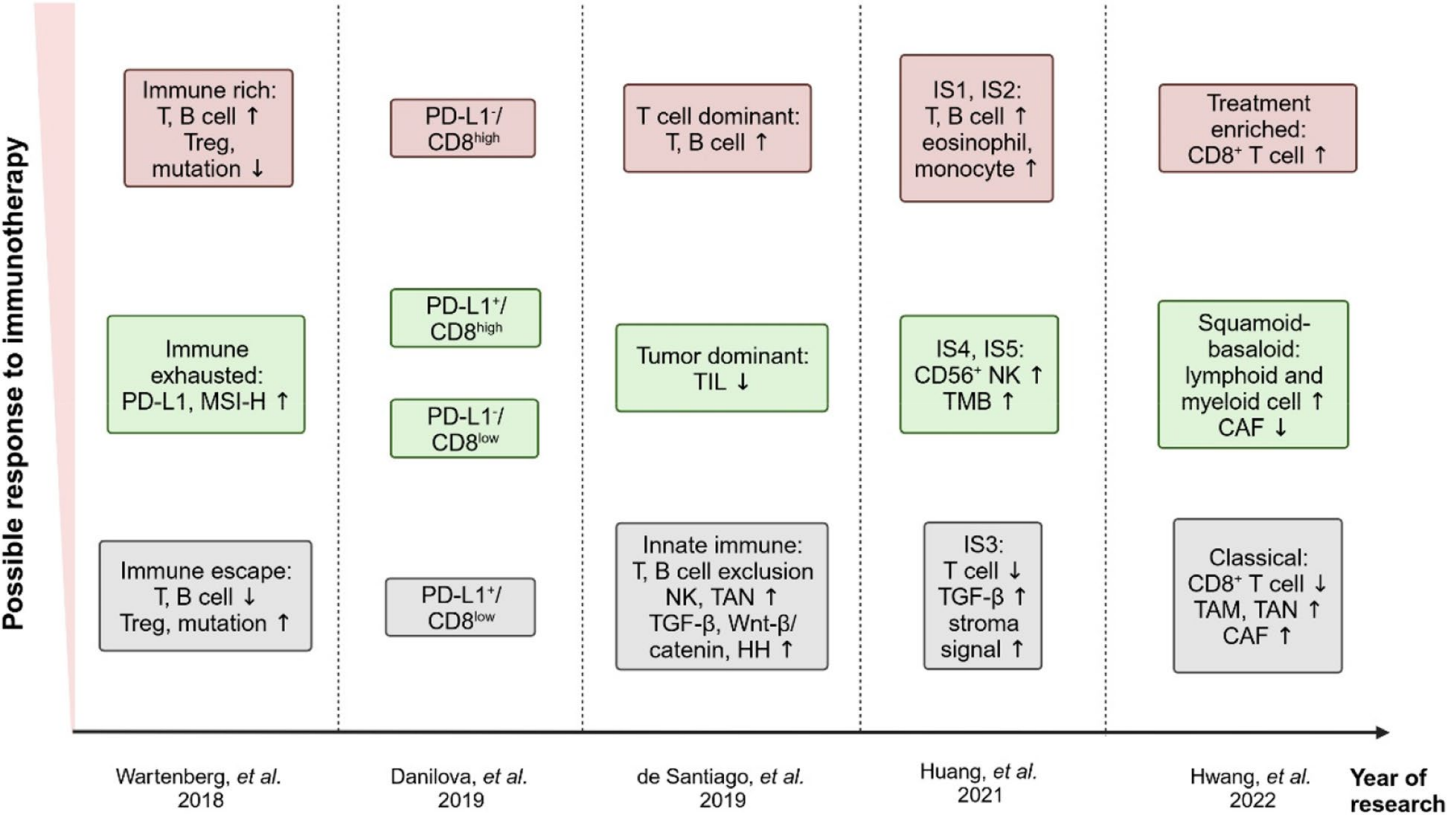
Research progress in immunophenotyping of PDAC. MSI: microsatellite instability; TIL: tumor infiltrating lymphocyte; TAN: tumor‐infiltrating neutrophil; HH: hedgehog signaling; TMB: tumor mutational burden; CAF: cancer-associated fibroblast; TAM: tumor-associated macrophage

The extensive infiltration of highly heterogeneous cancer-associated fibroblasts (CAF) and the accumulation of various extracellular matrix (ECM) components in the TIME are key characteristics that distinguish PDAC from other tumors. Notably, recent studies have significantly deepened our understanding of the roles of CAFs and ECM in influencing PDAC progression. Therefore, the concept of subtyping based solely on tumor and immune cells has proven insufficient to meet the precision demands of immunotherapy (Fig. 3). In 2021, Huang and colleagues analyzed RNA-seq data in PDAC samples and clustered immune related genes, identifying five immune subtypes (IS1–IS5). Eosinophils, activated CD8^+^ T cells, activated B cells, monocytes, and effector memory CD4^+^ T cells were more numerous in IS1 and IS2. Therefore, IS1 and IS2 were correlated with better survival, and there is hope for better responses of ICIs [128]. In IS4 and IS5, tumors were immunologically cold and associated with higher TMB, while fewer T cells and more CD56^+^ NK cells were found. Therefore, this study proposed the feasibility of using mRNA vaccines to treat IS4 and IS5 subtypes. Compared to traditional cancer vaccines, mRNA vaccines pose no risk of insertional mutagenesis, have a short and regulatable in vivo half-life, representing a safer and more personalized precision-therapy. These mRNA vaccines are poised to enhance antigen presentation and drive the expansion of tumor-specific T cells, with the aim of reversing the immunosuppressive TIME [129]. It is worth noting that IS3, as a high-matrix phenotype, is associated with the worst survival. The characteristic of IS3 is minimal infiltration of immune cells, accompanied by high up-regulation of TGF-β and stroma signaling [128]. Consequently, a combined stroma-targeted therapy becomes imperative for the IS3 subtype. A recent study constructed the spatial structure and immune landscape of PDAC. The researchers identified 14 malignant cell programs that reflected either lineage (classical, squamoid, basaloid, mesenchymal, acinar-like, neuroendocrine-like and NRP) or cell state (cycling-S, cycling-G2/M, MYC, interferon, TNF-NF-κB, ribosomal, and adhesive) and four CAF programs (myofibroblastic progenitor, neurotropic, immunomodulatory, and adhesive) using single-nucleus RNA sequencing and whole-transcriptome digital spatial profiling of PDAC specimens [130]. Further, three multicellular communities with distinct immune signatures are revealed through unsupervised clustering. Community 1 (treatment enriched) was characterized by an association among the neural-like progenitor (NRP) and neuroendocrine-like malignant programs, neurotropic CAF program and CD8^+^ T cells, which were all enriched with treatment, as well as the mesenchymal and acinar malignant programs and the immunomodulatory CAF program. Community 2 (squamoid-basaloid) featured an association of the squamoid and basaloid malignant programs with a diverse set of lymphoid and myeloid cell types, higher epithelial and immune content and lower CAF content. Community 3 (classical) exhibited an association among the classical malignant program, the myofibroblastic progenitor and adhesive CAF programs, macrophages, neutrophils, and cDC2s, as well as higher CAF and lower immune proportions [130]. This new community classification demonstrates how different tumor cell subtypes evolve in a coordinated manner with various components of TIME during PDAC progression. Moreover, it incorporates programs, not just cellular components, into the evaluation of subtyping. Different subtypes correspond to different CAF features and related signal markers. Among the four CAF programs, the ACTA2-enriched myofibroblastic progenitor program overlapped with a myofibroblastic CAF signature, while the neurotropic, immunomodulatory, and adhesive programs all overlapped with the single-cell inflammatory CAF signature. Based on this, reasonable targeting methods can be added based on stroma expression of different subtypes to achieve stroma normalization of TIME. In the following section, we summarize CAF and ECM components, as well as the corresponding targeted therapies.

### CAF: the most characteristic cell in the TIME

#### Heterogeneity and subtypes

**Fig. 4:**
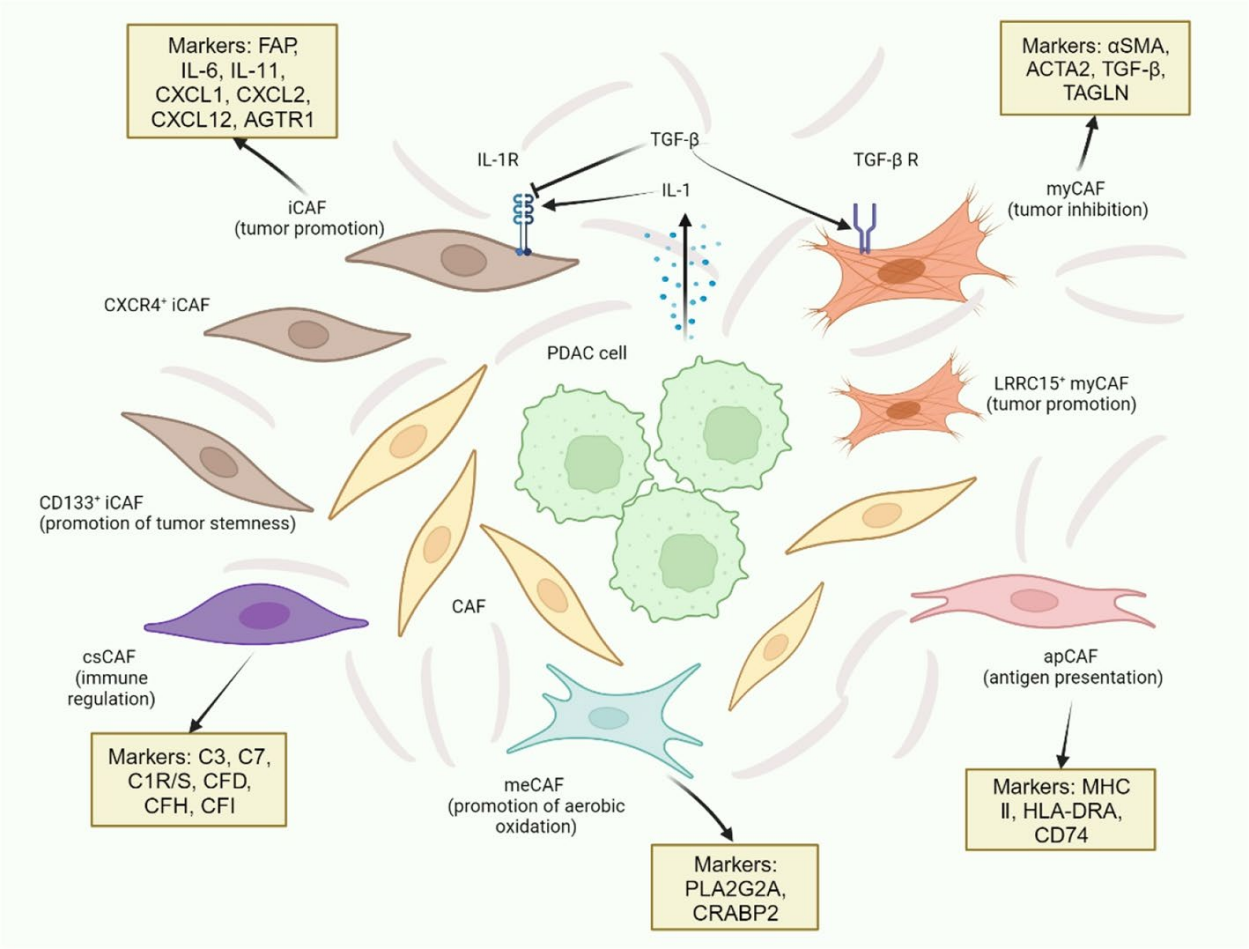
Various CAFs in PDAC, CAF function, and plasticity. CAFs in the TIME of PDAC are mainly divided into iCAFs, myCAFs and apCAFs, which perform different functions. These three classical subtypes can be further subdivided by different markers. New subtypes, such as csCAFs and meCAFs, are also being discovered. Tumor cells can promote the activation of iCAF by upregulating the expression of IL-1, and TGF-β signaling can induce the proliferation of myCAF by antagonizing this process. This CAF plasticity suggests that we can achieve the purpose of treatment by reprogramming CAFs. CAF: cancer-associated fibroblast; iCAF: inflammatory CAF; myCAF: myofibroblastic CAF; apCAF: antigen-presenting CAF; LRRC15: leucine-rich repeat containing 15; csCAF: complement-secreting CAF; meCAF: CAF with a highly activated metabolic state

The highly heterogeneous CAFs in PDAC infiltrate in large numbers, which are believed to be derived from PSCs, tissue resident fibroblasts, mesenchymal stem cells, and endothelial cells [131]. Therefore, CAFs are accordingly categorized into multiple subtypes (Fig. 4). Previous studies have identified three classical subtypes of CAFs: a) inflammatory CAFs (iCAF), characterized by low α-smooth muscle actin (αSMA, gene name *ACTA2*) expression, up-regulation of JAK/STAT signaling and high expression of cytokines (*IL-6*, *IL-11*) and chemokines (*CXCL1*, *CXCL2*); b) myofibroblastic CAFs (myCAF), with up-regulation of *ACTA2* and TGF-β response genes; and c) antigen-presenting CAFs (apCAF), which express MHC II molecules but lack the co-stimulatory molecules CD80 and CD86 required to induce T-cell proliferation [132–134]. The characteristic markers of these three important CAF subtypes are still being explored. For instance, Zhou and colleagues have found that *TAGLN* and *ACTA2* discern myCAFs, fibroblast activation protein (*FAP*) and *CXCL12* distinguish iCAFs, and apCAFs express *HLA-DRA* and *CD74* [56]. Furthermore, these three classical CAF subpopulations may be subdivided by novel markers. For example, CXCR4^+^ iCAFs and CD133^+^ iCAFs were observed in iCAFs, in which CD133^+^ iCAFs expressed cancer stem cell markers, including *CD133*, *MET*, *EPCAM*, *CD24* and *CD44* [56]. A single-cell RNA sequencing identified a subpopulation of myCAFs that are programmed by TGF-β and express the leucine-rich repeat containing 15 (LRRC15) protein. These LRRC15^+^ CAFs are absent from normal pancreatic tissue and correlated with poor response to anti-PD-L1 therapy [135]. After selectively depleting LRRC15^+^ CAFs, the CAF composition could be recalibrated towards universal fibroblasts in a mouse model, accompanied by the recovery of anti-tumor immunity [136]. Another study used mass cytometry to divide CAFs into two populations based on the expression of CD105. CD105^+^ CAFs allow tumor growth in vivo, while CD105^−^ CAFs are highly tumor suppressive [137]. Interestingly, CD105^−^ CAFs express some markers of apCAFs and mesothelial cells (MHC II and CD74), and determination of their origin requires further lineage tracing studies [137]. Elucidating the full spectrum of CAF heterogeneity in PDAC remains in its early stages. With ongoing advances in single-cell sequencing technologies in recent years, new CAF subtypes are being continuously discovered. For example, a study identified a complement-secreting CAFs (csCAF) that specifically expresses complement components C3, C7, C1R/S, CFD, CFH, CFI [57]. However, csCAFs shared similarities with iCAFs in that the latter also exhibited abundant expression of C3 and CFD. Therefore, further experiments are needed to clarify whether the differences in the two types of cells are due to the heterogeneity of the tumor samples. In another study, a novel subtype of CAFs with a highly activated metabolic state (meCAFs) was found in loose-type (low desmoplasia) PDAC compared to dense-type (high desmoplasia) PDAC. MeCAFs have highly active glycolysis and produce large amounts of metabolic intermediates as a fuel source for mitochondrial OXPHOS in cancer cells [138]. A higher amount of meCAFs is correlated with a higher risk of metastasis and a poor prognosis, while presenting a significantly better response to immunotherapy (64.71% ORR, one complete response) [138]. Thus, PDAC patients whose microenvironment is heavily enriched with meCAFs may benefit from a combination of ICIs and OXPHOS inhibitors. It is our ultimate purpose to make the subtyping of CAFs more concise. This requires precise isolation of CAF subpopulations and identification of their characteristic markers through single-cell sequencing to explore the potential functions of CAFs against PDAC. An improved understanding of CAF heterogeneity serves as the foundation for future personalized therapeutic regimens targeting CAFs.

#### Function and plasticity

Different CAF subpopulations play distinct roles during PDAC development. Generally, iCAFs have a tumor-promoting function and are responsible for chemoresistance, myCAFs are proposed to have a tumor-restraining role, and apCAFs have a putative immune-modulatory role involving antigen presentation [56, 139]. Recently, a marker-based study on CAF subtyping found that depletion of FAP^+^ CAFs results in increased survival, in contrast to depletion of αSMA^+^ CAFs, which leads to decreased survival [140]. Moreover, IL-6 deletion in αSMA^+^ CAFs improved gemcitabine efficacy and synergized with checkpoint blockade therapy, illustrating the potential immunosuppressive effects of αSMA^+^ CAFs [140]. But this population of cells is not fully equivalent to classical myCAFs, and the extent of overlap between the two should be explored in greater depth.

Both iCAFs and myCAFs possess the potential to transition into each other. IL-1 could activate downstream JAK/STAT signaling and mediate the induction of iCAFs, whereas TGF-β antagonizes this process by down-regulating IL-1R1 expression and promoting the differentiation of myCAFs [141]. Another study identified alterations in CAF subsets during the progression of PDAC over time. myCAFs were well represented in high-grade intraductal papillary mucinous neoplasms, whereas iCAFs were found only in invasive cancer specimens [142]. This observation aligns with the concept that the microenvironment becomes increasingly immunosuppressive as PDAC advances. In addition, there are in vivo findings showing that genetic depletion of the transcription factor *Prrx1* could affect CAF plasticity. *Prrx1*-expressing CAFs stimulate hepatocyte growth factor signaling, thereby promote epithelial mesenchymal transition (EMT) of PDAC cells [143]. PDAC patients with high stromal expression of *Prrx1* display the most aggressive subtype. Whereas *Prrx1*-deficient CAFs express more αSMA and appear to be converted towards the myCAF subtype. This CAF phenotype secretes matrix proteins that support stromal expansion and inhibit tumor dissemination [143]. By further comprehending the role of regulatory factors in CAF plasticity, we may be able to strategically shape CAFs to adopt a tumor-suppressive phenotype, potentially enhancing the efficacy of PDAC treatment.

#### CAF signaling: continuously modeling the immunosuppression of the TIME

**Fig. 5:**
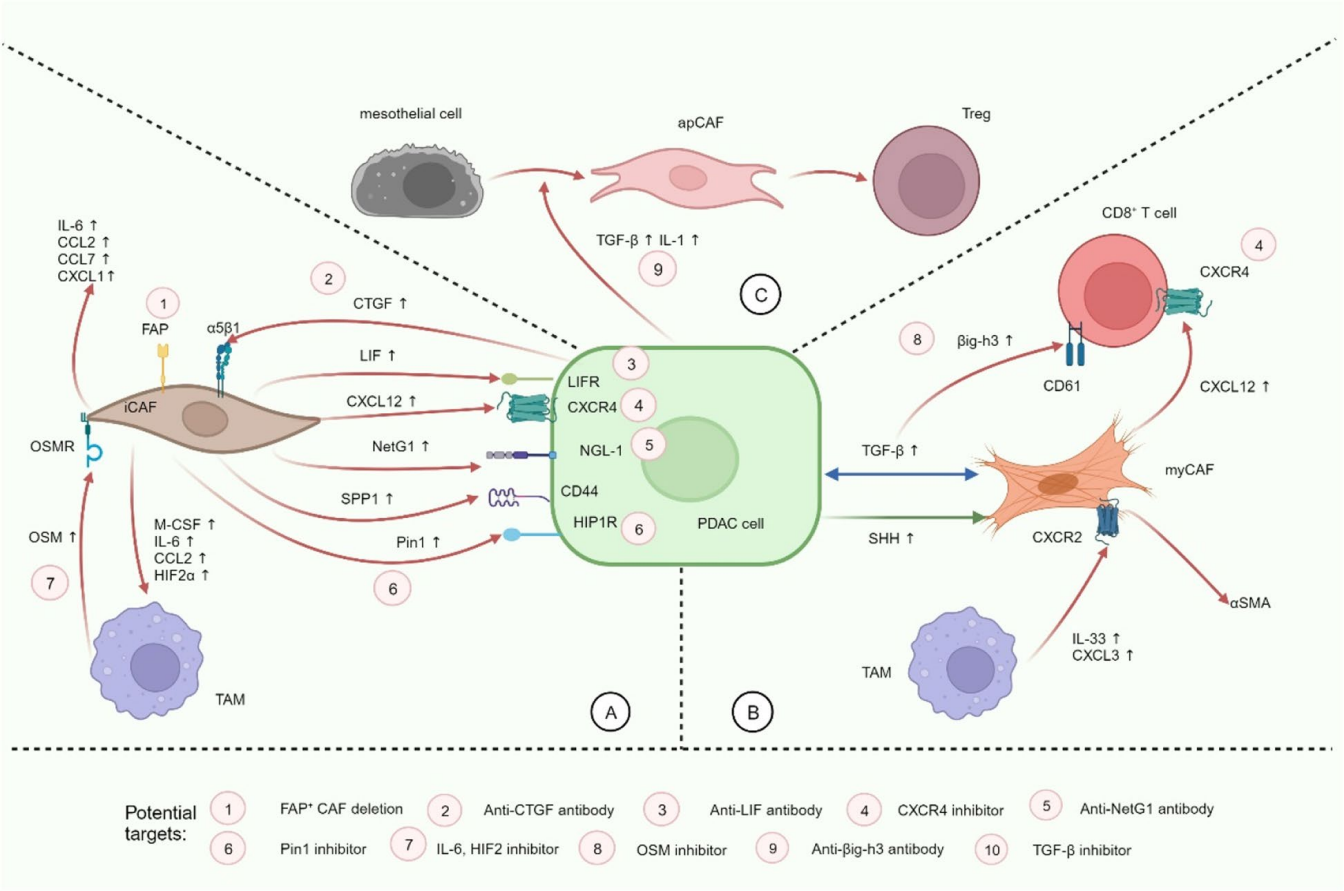
Signaling of three important CAFs in TIME of PDAC. **A** Tumor cells promote the activation of iCAFs through CTGF and so on. Activated iCAFs release a large number of factors that act on receptors such as LIFR, CXCR4, NGL-1, CD44, and HIP1R on tumor cells to promote proliferation, metabolism, metastasis, and stemness. iCAFs promote the proliferation and M2 polarization of TAMs by releasing a variety of factors. TAMs can promote iCAFs to release more tumor-promoting factors through OSM-OSMR. **B** Tumor cells upregulate SHH signaling to recruit tumor-suppressing myCAFs. Both tumor cells and myCAFs can promote the proliferation of each other by upregulating TGF-β signaling. myCAFs show tumor suppression or tumor promotion after being activated by TGF-β, which may depend on the time of tumor progression. TGF-β and CXCL12 act on the corresponding receptors on CD8^+^ T cells to mediate their exhaustion. **C** Tumor cells promote the transformation of mesothelial cells into apCAFs through TGF-β and IL-1, thereby inducing the proliferation of Tregs. Red arrows represent tumor-promoting processes, green arrows represent tumor-inhibiting processes, blue arrows indicate that the process of promoting or inhibiting tumor has not yet been determined. Pink circles represent possible treatment strategies. iCAF: inflammatory CAF; FAP: fibroblast activation protein; CTGF: connective tissue growth factor; LIF: leukemia inhibitory factor; NetG1: Netrin G1; SPP1: secreted phosphoprotein 1; OSM: oncostatin M; Pin1: peptidylpropyl isomerase; TAM: tumor-associated macrophage; myCAF: myofibroblastic CAF; SHH: sonic hedgehog; αSMA: α-smooth muscle actin; apCAF: antigen-presenting CAF

The exploration of the interactions between CAFs and various cell components within the PDAC microenvironment is an emerging area of research with significant potential. Broadly, CAFs are activated by diverse signals in the microenvironment, which in turn nourish tumor cells and drive immune cells towards a more immunosuppressive phenotype by secreting various immunosuppressive factors. This intricate network of malignancy fosters both the growth of PDAC and its evasion from immune surveillance (Fig. 5). During this process, heterogeneous CAF populations demonstrate diverse functions, some of which may even display tumor suppressive characteristics.

Interaction with cancer cells: Previous studies indicated that PDAC cells can induce adjacent quiescent fibroblasts to become CAFs by activating paracrine sonic hedgehog (SHH) signaling and CXCL12/CXCR4 signaling, and by secreting diverse inflammatory cytokines and growth factors, such as connective tissue growth factor (CTGF), TGF-β and IL-1 [74, 144, 145]. Increased release of leukemia inhibitory factor (LIF) and TGF-β has been found in activated CAFs and leads to activation of MEK/ERK and STAT3 signals to promote PDAC progression [146, 147]. CAFs function to promote stemness features of tumor cells. Long term treatment of CAF-conditioned media induced PDAC cells to exhibit stemness features via significant up-regulation of the osteopontin/secreted phosphoprotein 1 (SPP1)-CD44 axis [148]. CAFs also support PDAC metabolism. Koikawa and colleagues found peptidylpropyl isomerase (Pin1) over-expressed in CAFs, which promotes oncogenic signaling pathways and reduces the expression of PD-L1 and the gemcitabine transporter ENT1 on the PDAC cell surface [149]. When Pin1 was inhibited, the release of immunosuppressive factors from cancer cells and CAFs could be suppressed, and the number of tumor-infiltrating CD8^+^ T cells was increased [149]. The findings suggest a potential therapeutic strategy, using Pin1 inhibitors in combination with αPD1 and gemcitabine to eradicate invasive PDAC. In addition, a study identified up-regulation of the glutamatergic pre-synaptic protein Netrin G1 (NetG1) in CAFs in a mouse model. These immunosuppressive NetG1^+^ CAFs enable PDAC cells to survive under low-nutrient conditions and reduce NK cell-induced death by releasing glutamate and glutamine [150]. Knockdown of NetG1 reverses pro-tumorigenic CAF markers and reduces PDAC cell survival [150].

Interestingly, previous studies have also found the potential of certain CAFs to suppress PDAC progression, which express αSMA and are activated by paracrine SHH signaling from tumor cells [74, 151]. It is becoming evident that this particular group of CAFs might correspond to myCAFs or represent a distinct, yet unidentified, subgroup of CAFs with characteristics akin to myCAFs. Therefore, as we move forward, it becomes imperative to design experiments that build upon our comprehension of CAF heterogeneity. This will enable us to delve deeper into the intricate influences wielded by various CAF subpopulations on the growth of PDAC cells.

Interaction with other microenvironment components: During PDAC progression, CAFs exert long-lasting educative effects on the surrounding immune cells, with the overall effect of inhibiting their normal function and inducing a shift towards a pro-tumorigenic phenotype. Previous studies have shown that CAF can promote the differentiation and activation of M2 macrophages through M-CSF, IL-6, and CCL2 [131]. HIFs mediate the cellular response to hypoxia. Mechanistically, CAFs promoted macrophage Arg-1 expression and polarization towards M2 phenotype in a HIIF2-dependent paracrine manner. CAF-specific deletion of *Hif2α*, but not *Hif1α*, could suppress PDAC tumor progression and growth, and improved survival of mice by 50% [152]. Knockout of CAF-HIF2 reduced tumor fibrosis and the recruitment of immunosuppressive M2 macrophages and regulatory T cells [152]. Similarly, TAMs can reciprocally impact CAFs, thereby fostering desmoplasia. A recent study showed that oncostatin M (OSM) secreted by macrophages stimulated CAFs with high expression of IL-6, CCL2, CCL7, and CXCL1 via OSMR [153]. Whereas Osm-deficient mice exhibit increased abundance of αSMA^+^ myCAFs and reduced tumor growth and metastasis [153]. OSM also acts as an inducer of lysyl oxidase-like protein 2 expression, which is responsible for collagen and elastin cross-linking. This matrix remodeling promotes EMT and stemness of PDAC and reduces OS [154]. Sun and colleagues discovered that TAM-CAF regulated PDAC metastasis through interaction along the IL-33-ST2-CXCL3-CXCR2 axis. Mechanistically, CXCL3 was highly up-regulated in IL-33-stimulated macrophages, while its receptor CXCR2 was almost exclusively expressed in CAFs [155]. CXCL3-CXCR2 signaling induced CAF to myCAF transition, which is accompanied by the up-regulation of αSMA. Type III collagen was identified as the CXCL3-CXCR2-targeted adhesive molecule responsible for myCAF-driven PDAC metastasis [155]. myCAFs are generally tumor suppressive, but a surprising result achieved in this study showed that myCAFs were found to hijack PDAC cells for metastasis. It is possible that myCAFs acquire this ability after macrophage education, or, a subset within myCAFs is intrinsically tumor-promoting and needs to be further identified on the basis of markers.

CAFs could influence tumor immunity through the communication with T cells. CXCL12 secreted by CAFs can recognize CXCR4 on the surface of T cells. The researchers found that this CXCL12-CXCR4 axis could lead to a reduction in the number of infiltrated T cells and exacerbate immunosuppression of TIME [156, 157]. In addition, Goehrig and colleagues found that TGF-β could promote myCAFs to produce a matrix protein, βig-h3, which could interact with CD61 on the surface of CD8^+^ T cells and macrophages [158]. Depleting βig-h3 in vivo reduced tumor growth by enhancing the number of activated CD8^+^ T cell within the tumor and subsequent apoptotic tumor cells [158]. We previously introduced apCAFs overexpressing MHC II molecules, which one study found to be actually derived from mesothelial cells. During PDAC progression, mesothelial cells form apCAFs by down-regulating mesothelial features and gaining fibroblastic features, a process induced by IL-1 and TGF-β [159]. apCAFs directly induce naive CD4^+^ T cells into Tregs in an antigen-specific manner. In sum, a multitude of further experiments are warranted to elucidate the intricate mechanisms underlying CAF functions in PDAC. The therapeutic potential of targeting CAFs in the treatment of PDAC is two-pronged: it aims not only to eliminate the subset of CAFs that promote tumor progression, along with their secreted tumor-promoting factors, but also to alleviate immunosuppression within TIME through modulation of interactions with immune cells.

#### Targeting CAFs: CAF normalization

CAF-targeted therapies mainly have three directions: a) deleting CAFs, b) inhibiting the tumor-promoting signaling of CAFs, and c) reprogramming CAFs. Directly deleting CAFs based on their marker expressions represents a “simple and crude” approach, especially in the case of FAP^+^ CAFs that lead to poor prognosis. FAP, which leads to a poor prognosis, is more often studied at present. FAP-based DNA vaccines can induce FAP-specific tumor-infiltrating lymphocytes to specifically clear CAFs and disrupt tumor tolerance [160]. FAP is also one of the important targets for CAR-T cell therapy. A recent study developed a radio-labelled FAP inhibitor probe, which was able to track FAP expression on tumor cells and stromal cells in the TIME with a high target-to-background ratio. This FAP probe has the potential to predict and monitor the efficacy of FAP-targeted CAR T-cell therapy [161]. Notably, FAP is not exclusively expressed on CAFs, implying that the toxicity associated with the deletion of FAP^+^ cells needs to be addressed in this approach. This also underscores the necessity for the identification of more selective markers to enhance the precision of CAF-depletion therapies. Inhibition of the tumor promoting signal released by CAF seems to be a more eagerly anticipated approach.

CXCR4 antagonists AMD3100 and BL-8040 (motifafortide) have been found to slow PDAC growth by inhibiting CAFs [162, 163]. In a subsequent phase II study, triple therapy with motifafortide, pembrolizumab and chemotherapy showed signs of efficacy in the poor prognosis and aggressive PDAC population, with a median PFS of 3.8 months and a median OS of 6.6 months [164]. In addition, Zhou and colleagues found that E26 transformation-specific homologous factor could decrease the sensitivity of PDAC cells to niche stimulus of cancer stem cells by inhibiting the transcription of CXCR4 [165]. Xie and colleagues developed a CXCR4 antagonist nanoparticle, and they found that its combination with miR-210/KRAS^G12D^ blockade increased matrix consumption, resulting in decreased immunosuppression and delayed tumor growth [166]. This study raised the possibility of using nanoparticles as carriers for delivering CAF modulators to enhance their effectiveness. TGF-β, IL-6/STAT3 are also important signals in the process of CAF activation and function. Direct inhibition of TGF-β, blocking its downstream signaling and antagonizing its receptor can reverse the activated CAFs [167–169]. Given the role of mutually activated MEK and STAT3 pathways in mediating PDAC development, one study employed MEK inhibitor (trametinib) and STAT3 inhibitor (ruxolitinib) in a mouse model. MEKi + STAT3i attenuated IL-6/CXCL1-expressing proinflammatory and LRRC15-expressing myCAFs while enriching Ly6a/CD34-expressing CAFs exhibiting mesenchymal stem cell-like features [170]. Meanwhile, this combination therapy is associated with M2-to-M1 reprogramming of macrophages, enrichment of CD8^+^ T cells, and helps PDAC patients overcome the resistance of PD-1 inhibitors [170].

Reprogramming CAFs according to their plasticity represents an innovative direction. Our objective is to convert the tumor promoting CAF phenotype to a tumor-suppressive phenotype or to revert activated CAFs to a quiescent phenotype. This is a more appealing approach due to its ability to recapitulate the normal CAF state in the microenvironment. Approaches targeting IL-1 and TGF-β have the potential to yield additional therapeutic effects due to their effects in transforming CAFs. The means of activating TGF-β seem to be counterintuitive, even though previous studies found it able to suppress the iCAF phenotype. The timing of drug delivery must be considered, as TGF-β may only exhibit tumor-suppressive functions in the early stages of PDAC. Studies have also found vitamin A analogues and the activation of vitamin D receptors to convert CAFs into a quiescent state before their activity [131]. Agonists of vitamin D receptor are promising in the future for PDAC treatment, and all-trans retinoic acid has shown some therapeutic efficacy in a phase I trial [171, 172]. Additionally, the exploration of CAF-targeted approaches involving Minnelide, lipoxin a4, and serum amyloid A1 is ongoing [173]. Our concept is constantly being updated, that is, from deleting CAFs to remodeling CAFs. Building on a deeper comprehension of CAF subtypes, markers, and plasticity, we may be better placed to target CAFs with precision and restore a balanced number and function. This approach holds the potential not only to restrain PDAC progression but also to minimize potential toxic side effects as much as possible throughout the process.

### ECM

#### ECM signaling: a double-edged sword

**Fig. 6:**
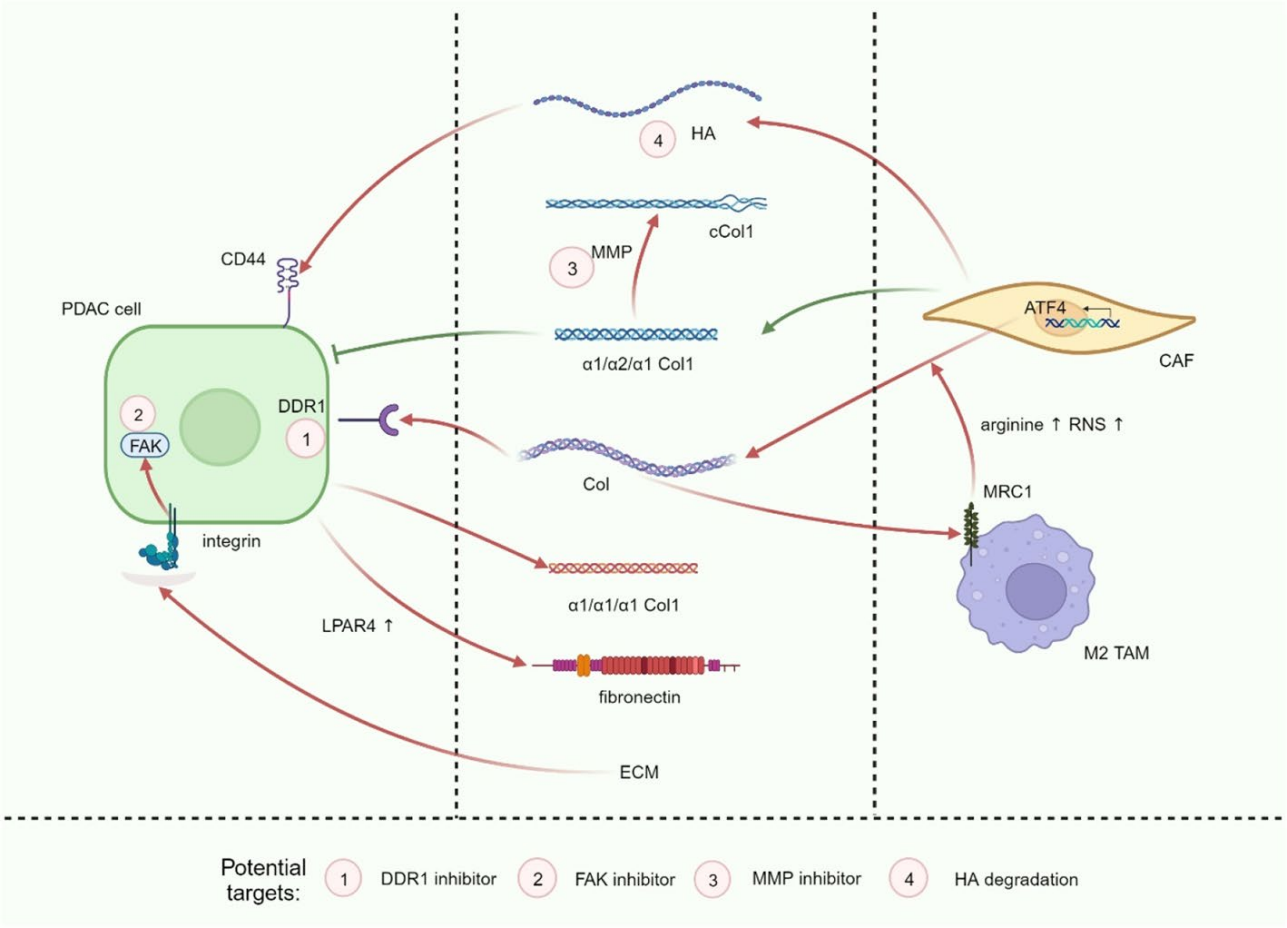
Interactions between PDAC cells and the ECM. Collagen (Col) is mainly produced by CAFs and acts on DDR1 to promote tumor proliferation. Col activates MRC1 on the surface of TAMs, which further promotes its production by upregulating ATF4 within CAFs, forming a vicious cycle. In contrast to the tumor-suppressing heterotrimeric Col1 produced by CAFs, tumor cells can produce tumor-promoting homotrimeric Col1. MMP can cleave col1 to produce tumor-promoting cCol1. HA produced by CAFs accumulates massively in the microenvironment and mediates growth-promoting signals by acting on CD44. FAK signaling is activated by integrins and mediates connective tissue formation, immunosuppression, and metastasis. Moreover, tumor cells induce the expression of fibronectin to promote therapeutic resistance. Red arrows represent tumor-promoting processes. Pink circles represent possible treatment strategies. DDR1: discoidin domain receptor 1; FAK: focal adhesion kinase; HA: hyaluronan; Col1: collagens I; MMP: matrix metalloproteinase; LPAR4: lysophosphatidic acid receptor 4; ECM: extracellular matrix; CAF: cancer-associated fibroblast; ATF4: activating transcription factor 4; MRC1: mannose receptor C-type 1; RNS: reactive nitrogen species; TAM: tumor-associated macrophage

The ECM of PDAC demonstrates two characteristics: a) massive accumulation and cross-linking of stromal components, and b) expression of multiple tumor-promoting signals (Fig. 6). Collagen and hyaluronan are the predominant constituents of the ECM in PDAC, and their continuous accumulation leads to a progressive “hardening” of the ECM, with elevated pressure and impaired perfusion. This phenomenon poses dual challenges: on the one hand, this hinders the infiltration of immune cells and the delivery of drugs to deeper tumor tissues; on the other hand, elevated pressure between tumor niches and margins accelerates tumor invasion to surrounding tissues and metastasis to distant organs. Meanwhile, these stromal alterations in PDAC mediate an increase in tension and contractility of the adjacent epithelium, accelerating tumor growth and shortening survival [174].

Collagens in PDAC are mainly collagens I (Col1), III and IV, which mediate a variety of signals influencing tumor progression. The process of collagen synthesis by CAFs is controlled by activating transcription factor 4 (ATF4), a major effector of the integrated stress response [175]. Traditionally, collagen is believed to promote tumors and correlated with poorer prognosis [176]. Mechanistically, collagen can bind to discoidin domain receptor 1 (DDR1) and promote tumor cell growth and migration [174, 177]. In addition, collagen internalization mediated by mannose receptor C-type 1 (MRC1) could lead to the accumulation of arginine within M2 type TAMs, which promoted the production of reactive nitrogen species (RNS) [178]. PSCs exposed to RNS enhanced pancreatic intra-tumoral fibrosis and increased collagen deposition in vivo, resulting in a vicious cycle [178]. However, one study found a role for fibrillar collagen derived from cancer cells in selective inhibition of PDAC growth, suggesting that specific components within collagen might possess yet-unexplored anti-tumor properties [179]. Recent studies have advanced our understanding of Col1. Activated PSCs/αSMA^+^ myCAFs are major contributors to Col1 in PDAC stroma and have a tumor-suppressive function. A decrease in Col1 content could lead to the up-regulation of *Cxcl5* in cancer cells via SOX9, and was associated with the recruitment of MDSCs and suppression of CD8^+^ T cells [180]. In contrast to normal Col1 heterotrimer (α1/α2/α1) produced by CAFs, PDAC cells could specifically produce unique Col1 homotrimer (α1/α1/α1). Col1 homotrimers can enhance capacity for proliferation, tumor growth, and resistance to gemcitabine via DDR1 and integrin α3β1, and resulted in a tumor microbiome rich in anaerobic *Bacteroidales* [181]. Knockout of the Col1 gene of tumor cells in a mouse PDAC model resulted in the loss of Col1 homotrimers, which enhanced T cell infiltration and improved the efficacy of anti-PD-1 immunotherapy [181]. A recent study by Su and colleagues has shown that matrix-metalloprotease-cleaved Col1 (cCol1) and intact Col1 (iCol1) exert opposite effects on the growth and metastasis of PDAC. Mechanistically, Col1 acts through DDR1–NF-κB–p62–NRF2 to regulate tumor growth and metabolism, a cascade that is activated by cCol1 and inhibited by iCol1 [182]. Patients whose tumors are enriched for iCol1 and express low levels of DDR1 and NF-E2-related factor 2 (NRF2) have improved median survival compared to those whose tumors have high levels of cCol1, DDR1 and NRF2 [182]. It is suggested that Col1 remodeling serves as a prognostic indicator for survival in patients with PDAC.

In addition to collagen, the ECM contains a plethora of components teeming with signals that interact with tumors. Hyaluronan (HA), which is mainly over-expressed by PDAC cells and CAFs, has been found to bind mainly to the CD44 receptor on the surface of tumor cells and enhance tumor survival, proliferation, migration, and invasion through downstream signals such as MAPK, PI3K, and focal adhesion kinase (FAK) [183]. FAK is a non-receptor protein tyrosine kinase that can be activated by integrins on the surface of tumor cells [177]. As a poor prognostic indicator, FAK was able to drive desmoplasia, immunosuppression, and metastasis of PDAC by regulating the expression of matrix metalloproteinases (MMP), PI3K/AKT, and other signals [184, 185]. A recent study uncovered that PDAC cells exposed to environmental stress or chemotherapy could induce fibronectin expression by up-regulating lysophosphatidic acid receptor 4 (LPAR4), promoting an ECM-enriched niche and therapeutic resistance [186]. MMPs, over-expressed mainly by CAFs, are thought to lead to massive breakdown of the ECM, neovascularization, tumor spread, and metastasis. During PDAC progression, MMPs can be induced by Rho-associated protein kinases (ROCK) signaling to be further up-regulated [177]. Many studies have shown that SHH signal imbalance can promote the progress of PDAC by affecting the matrix. SHH signaling occurs in a paracrine mechanism in which hedgehog ligands produced by cancer cells bind to CAF receptors (such as Smoothened). This interaction enables CAFs activation, leading to cancer cell expansion, invasion, survival, and increased chemoresistance [174].

#### Targeting ECM: ECM normalization

One primary objective of targeting the ECM of PDAC is to deplete stromal components to alleviate vascular pressure and enhance drug delivery. Targeted formulations of collagen have great potential to benefit PDAC patients. Zinger and colleagues developed a collagenase nanoparticle called “collagozome”, which increased drug penetration and improved PDAC treatment in mice [187]. A crucial consideration pertains to whether it is feasible to achieve precise targeting of specific collagen types to spare those that confer benefits. As previously discussed, the generation of Col1 homotrimers by PDAC cells constitutes a “culprit” mechanism in tumor promotion. Alternatively, blockade of its receptor DDR1 and downstream signaling would have better efficacy. Pegvorhyaluronidase alfa (PEGPH20) was designed to degrade HA and thereby improve the delivery of chemotherapy drugs. However, the results of clinical trials targeting PEGPH20 were disappointing, with no concomitant improvement in survival [188–190]. The failure of PEGPH20 may be attributed to its persistent high-dose administration, potentially leading to reduced exposure to chemotherapy drugs, or it might stem from unforeseen negative drug interactions. Additionally, it is imperative to account for the heterogeneity of the stroma. For instance, HA-targeted drugs may be beneficial in regions of high-HA density but detrimental at sites of low-HA density. Chen and colleagues developed a gemcitabine@nanogel system consisting of a reduction-sensitive core, the payloads of gemcitabine, and the coronal of hyaluronidase. They confirmed its improvement of intra-tumoral penetration and anti-tumor efficacy in rats, which highlights that tailoring the mode of drug delivery has the potential to improve the efficacy of targeting HA [191]. Interestingly, studies have found that the angiotensin II receptor inhibitor losartan could decrease the content of Col1 and hyaluronan. Total neoadjuvant therapy with FOLFIRINOX, losartan, and chemoradiotherapy provided downstaging of locally advanced PDAC and was associated with a margin-negative resection rate of 61% [192].

FAK inhibitors can inhibit tumor adhesion to ECM [193]. However, FAK inhibition can lead to the relative activation of other immunosuppressive signals, underscoring the importance of combination therapy [194]. An illustrative study applied a combination therapy of FAK inhibitor, PD-1 inhibitor, and PEGPH20 to PDAC mice. This therapy was found to increase T cell infiltration and change the T cell phenotype to effector memory T cells. Concomitantly, the number of MDSC cells and CXCR4-expressing granulocytes decreased [195]. Recently, Wang-Gillam and colleagues completed a phase I trial of defactinib (a highly potent oral FAK inhibitor), pembrolizumab in combination with gemcitabine in the treatment of pancreatic cancer. The combination was well-tolerated and safe, and exhibited preliminary efficacy [196]. In addition, there is a study demonstrating that FAK inhibition could rescue the efficacy of radiotherapy, leading to tumor regression, T-cell priming, and enhanced long-term survival in PDAC mouse models [197]. The combination of FAK inhibitors with chemoradiotherapy, immunotherapy is expected to exhibit certain benefits in subsequent clinical trials. Exploration targeting other components of the ECM, such as the ROCK inhibitor AT13148, is also ongoing [198]. Regrettably, the results of clinical trials of MMP inhibition (such as marimastat and tanomastat) and SHH inhibition (such as vismodegib, saridegib and IPI-926) have been largely disappointing, in part because of the complexity of the TIME and the contradictory outcomes that SHH signaling has produced in different models to either promote or inhibit tumors [173, 199, 200]. These outcomes provide valuable insights: a non-specific targeting approach directed solely at the ECM may not be efficacious in PDAC, and the hurried development of ECM-targeted agents without a comprehensive comprehension of the roles stromal signals play in PDAC progression could be counterproductive. It is conceivable that the inherent function of the ECM is to suppress tumor growth and metastasis. However, as PDAC advances, tumor cells reprogram the ECM to support their own growth. Consequently, the optimal timing for ECM-targeted therapies warrants further exploration. Collectively, studies targeting the ECM of PDAC are currently still in their preliminary stages. Our goal is the restoration of components and dysregulated signals to re-establish the initial tumor-suppressive state of the ECM. Before developing targeted drugs, a thorough clarification of their effects on PDAC and associated mechanisms is mandatory. Striving for a harmonized equilibrium within the ECM, achieved through the coordinated collaboration of multiple therapeutic modalities, holds the promise of delivering substantial benefits to patients.

### Endothelial cell: targeting angiogenesis

Angiogenesis in the TIME of PDAC is less than that of other solid tumors. The proliferative matrix accumulates in the TIME, resulting in high interstitial pressure and impaired perfusion, which leads to an extremely hypoxic environment. In previous years, clinical trials of PDAC targeting angiogenesis such as vascular endothelial growth factor (VEGF) blocking basically failed, partly because VEGF antibody is believed to induce tumor growth by exacerbating hypoxia [177]. Therefore, vascular normalization is a more effective strategy by pruning immature and inefficient endothelial cells to improve tumor perfusion. Previous studies have found that Semaphorin 3A secreted by endothelial cells participates in promoting physiological vascular normalization through the negative regulatory effect of integrins, which has the potential to serve as a target [177, 201]. A recent study found that, through upregulating the expression of the BICC1 gene, PDAC cells can activate the JAK2/STAT3 signaling and increase the production of the angiogenic factor CXCL1 [202]. Therefore, targeting this VEGF-independent angiogenesis has the potential to be combined with gemcitabine to improve the efficacy of PDAC, and further exploration is needed in clinical trials (Table 1) [202].

**Table 1:** Important clinical trials of TIME-targeted therapies that have achieved results in recent years

ID	Targeted strategies	Phase	Patients	Treatment	Main results
**Immune checkpoint inhibitor**					
NCT03404960 [31]	combined with PARP inhibition	Ib/II2	platinum-sensitive advanced pancreatic cancer (*n* = 91)	niraparib plus ipilimumab versus niraparib plus nivolumab	6-month PFS rate was 59.6% for niraparib plus ipilimumab, and 20.6% for niraparib plus nivolumab
NCT03565991 [32]		IIb	advanced BRCA1/2-altered or ATM-altered solid tumors (*n* = 200) including pancreatic cancer	avelumab and talazoparib	The confirmed ORR was 26.4% (42 patients, including 9 complete responses [5.7%]) in the BRCA1/2 cohort and 4.9% (2 patients) in the ATM cohort
NCT02309177 [33]	combined with chemotherapy	I	advanced pancreatic cancer (*n* = 50)	nivolumab, nab-paclitaxel, and gemcitabine	mPFS and mOS were 5.5 and 9.9 months, respectively
NCT02879318 [34]		II	metastatic PDAC (*n* = 180)	gemcitabine and nab-paclitaxel with or without durvalumab and tremelimumab	the combination immunotherapy did not improve survival among the unselected patient population (*p* = 0.72) immunotherapy group (*p* = 0.02)
NCT03104439 [36]	combined with radiotherapy	II	microsatellite stable PDAC (*n* = 25)	radiotherapy on ipilimumab plus nivolumab	DCR of patients was 20% (5 of 25; 95% CI, 7–41%), and reached 29% (5 of 17; 95% CI, 10–56%) after receiving radiotherapy
NCT02704156 [37]		II	locally recurrent pancreatic cancer after surgical resection (*n* = 170)	SBRT plus pembrolizumab and trametinib versus SBRT plus gemcitabine	mOS of 24.9 months (95% CI, 23.3–26.5) for SBRT plus pembrolizumab and trametinib, compared with 22.4 months (95% CI, 21.2–23.6) for SBRT plus gemcitabine (hazard ratio, 0.60; 95% CI, 0.44–0.82; *p* = 0·0012)
NCT02866383 [38]		II	refractory metastatic PDAC (*n* = 84)	SBRT/nivolumab/ipilimumab versus SBRT/nivolumab	clinical benefit rate of 37.2% (95% CI, 24.0–52.1%) for SBRT/nivolumab/ipilimumab versus 17.1% (95% CI, 8.0–36.0%) for SBRT/nivolumab
NCT02704156 [39]		II	PDAC patients characterized by mutant KRAS and positive immunohistochemical staining of PD-L1 and documented post-operative local recurrence (*n* = 170)	dose escalation of SBRT plus pembrolizumab and trametinib versus SBRT plus gemcitabine	SBRT/pembrolizumab/trametinib had longer OS compared with SBRT plus gemcitabine, but did not reach statistical significance (median: 15.1 vs. 12.4 months, HR 0.67 [95%CI 0.43–1.04]; *p* = 0.071)
NCT04258150 [40]	combined with IL-6 blockade and radiotherapy	II	refractory pancreatic cancer patients with intolerance to gemcitabine- or fluorouracil-containing regimens (*n* = 26)	pilimumab, nivolumab, and tocilizumab combined with SBRT	Five patients (19%; 95% CI, 7–39) achieved a stable disease. mPFS was 1.6 months (95% CI 1.4–1.7), and mOS was 5.3 months (95% CI 2.3–8.0)
NCT02734160 [60]	combined with TGF-β inhibitor	Ib	recurrent/refractory metastatic pancreatic cancer (*n* = 32)	galunisertib plus durvalumab	1 patient had partial response, 7 had stable disease, 15 had objective progressive disease. DCR was 25.0%. mOS and mPFS were 5.72 months (95% CI: 4.01 to 8.38) and 1.87 months (95% CI: 1.58 to 3.09)
NCT03098160 [203]	combined with prodrug that alleviates hypoxia	I	Advanced solid tumors (*n* = 22) including pancreatic cancer	evofosfamide plus ipilimumab	Of 18 patients with measurable disease at baseline, 3 (16.7%) achieved partial response and 12 (66.7%) achieved stable disease
**Other approaches targeting lymphocytes**					
NA [53]	TCR-T	NA	one patient with progressive metastatic pancreatic cancer	infusion of autologous T cells that clonally express two allogeneic HLA-C*08:02-restricted TCRs targeting mutant KRAS G12D	overall partial response rate was 62% at one month, and 72% at 6 month
NCT02436668 [70]	BTK inhibition	III	PDAC (*n* = 424) of stage IV diagnosis ≥ 6 weeks	ibrutinib plus nab-paclitaxel/gemcitabine (arm A) versus placebo plus nab-paclitaxel/gemcitabine (arm B)	no significant difference in OS between arm A versus arm B (median of 9.7 versus 10.8 months; *P* = 0.3225). PFS was shorter for arm A compared with arm B (median 5.3 versus 6.0 months; *P* < 0.0001). Overall response rates were 29% and 42%, respectively (*P* = 0.0058)
**Targeting myeloid cells**					
NCT01413022 [81]	CCR2 inhibition	Ib	borderline resectable or locally advanced PDAC (*n* = 47)	PF-04136309 plus FOLFIRINOX	16 of 33 patients receiving FOLFIRINOX plus PF-04136309 who had undergone repeat imaging achieved an objective tumor response, with local tumour control achieved in 32 patients
NCT02713529 [79]	CSF1R inhibition	II	Adult solid tumors (*n* = 116) including pancreatic cancer	AMG 820 plus pembrolizumab	safety profile was favorable but did not meet the predefined efficacy threshold
NCT02880371 [80]		Ib/II	Advanced solid tumors (19 in phase Ib, 57 in phase II) including PDAC	ARRY-382 plus pembrolizumab	One PDAC patient had partial response in phase Ib. One PDAC patient had a partial response lasting 2.4 months in phase II
NCT02588443 [85]	CD40 activatioin	I	resectable PDAC (*n* = 16)	selicrelumab	For selicrelumab-treated tumors, 82% were T-cell enriched, compared with 37% of untreated (*P* = 0.004) and 23% of chemotherapy/chemoradiation-treated (*P* = 0.012)
NCT03214250 [86]		Ib	untreated metastatic PDAC (*n* = 30)	Sotigalimab and gemcitabine plus nab-paclitaxel, with or without nivolumab	The combination was tolerable. Responses were observed in 14 of 24 DLT-evaluable patients
NCT03214250 [87]		II	metastatic PDAC (*n* = 105)	sotigalimab/nivolumab/chemotherapy	sotigalimab/nivolumab/chemotherapy arm did not show a meaningful improvement in 1-year OS rate (41.3%, *P* = 0.223, *n* = 35) compared to nivolumab/chemotherapy (57.7%, *P* = 0.006, *n* = 34) and sotigalimab/chemotherapy (48.1%, *P* = 0.062, *n* = 36)
NCT03013218 [95]	CD47 block	I	Advanced solid tumors (*n* = 110) including pancreatic cancer	Evorpacept alone and in combination with pembrolizumab or trastuzumab	Evorpacept in combination with pembrolizumab or trastuzumab exhibited safety and preliminary antitumor activity
NCT01896869 [125]	GVAX	II	metastatic PDAC (*n* = 82)	GVAX and ipilimumab	mOS was 9.38 months (95% CI, 5.0–12.2) for test group and 14.7 months (95% CI, 11.6–20.0) for control group (HR, 1.75; *P* = 0.019)
NCT02243371 [125]		II	metastatic pancreatic cancer (*n* = 93)	cyclophosphamide/GVAX followed by CRS-207 with (Arm A) or without nivolumab (Arm B)	mOS in Arms A and B were 5.9 (95% CI, 4.7–8.6) and 6.1 (95% CI, 3.5–7.0) months, respectively, with an HR of 0.86 (95% CI, 0.55–1.34)
NCT00727441 [128]		II	resectable PDAC (*n* = 87)	GVAX alone (arm A), GVAX/intravenous cyclophosphamide (arm B), GVAX/oral cyclophosphamide (arm C)	Arm A had a trend toward longer mOS (35.0 months) than that (24.8 months) in the historical controls. Arm C had a significantly shorter DFS than Arm A
NL7432 [116]	DC vaccine	I	resected PDAC (*n* = 10)	allogeneic lysate-DC vaccination	No vaccine-related serious adverse events were observed. Seven patients have not experienced disease recurrence or progression at a median follow-up of 25 months (15–32 months)
**Targeting CAFs**					
NCT03307148 [172]	ATRA	Ib	advanced, unresectable PDAC (*n* = 27)	gemcitabine, nab-paclitaxel and ATRA	mOS of 11.7 months (95%CI, 8.6–15.7)
NCT02826486 [164]	CXCR4 inhibition	II	metastatic PDAC (*n* = 37 in cohort 1, *n* = 22 in cohort 2)	BL-8040, pembrolizumab and chemotherapy	ORR is 21.1%, with confirmed ORR of 13.2%. mPFS is 3.8 months and mOS is 6.6 months. DCR of 34.5% in cohort 1, ORR and DCR of 32% and 77% in cohort 2
**Targeting ECM**					
NCT01839487 [189]	hyaluronan depletion	II	previously untreated metastatic PDAC (*n* = 279)	PEGPH20 plus nab-paclitaxel/gemcitabine (PAG) versus nab-paclitaxel/gemcitabine (AG)	median PFS is 9.2 months with PAG versus 5.2 months with AG (HR, 0.51; 95% CI, 0.26 to 1.00; *P* = .048)
NCT01959139 [191]		Ib/II	untreated metastatic PDAC (*n* = 138)	PEGPH20 plus mFOLFIRINOX versus mFOLFIRINOX	PEGPH20 plus mFOLFIRINOX has a significant increase in grade 3–4 toxicity without prolonging mOS (odds ratio, 2.7; 95% CI, 1.1 to 7.1)
NCT02715804 [190]		III	untreated, metastatic, hyaluronan-high PDAC (*n* = 494)	PAG versus AG	mOS of 11.2 months for PAG versus 11.5 months for placebo plus AG (HR, 1.00; 95% CI, 0.80 to 1.27; *P* = .97); mPFS is 7.1 months versus 7.1 months (HR, 0.97; 95% CI, 0.75 to 1.26])
NCT01821729 [193]	angiotensin II receptor inhibition	II	previously untreated locally advanced unresectable pancreatic cancer (*n* = 49)	FOLFIRINOX, losartan, and chemoradiotherapy	margin-negative resection rate of 61%
NCT02546531 [197]	FAK inhibition	I	Advanced treatment refractory pancreatic cancer (*n* = 20 in refractory cohort, *n* = 10 in maintenance cohort)	defactinib, pembrolizumab, and gemcitabine	mPFS and mOS were 3.6 and 7.8 months for refractory cohort, and 5.0 and 8.3 months for maintenance cohort

*PARP* Polyadenosine-diphosphate-ribose polymerase, *ATM* Ataxia telangiectasia mutated, *ORR* Objective response rate, *OS* Overall survival, *PFS* Progression-free survival, *DCR* Disease control rate, *SBRT* Stereotactic body radiotherapy, *BTK* Bruton's tyrosine kinase, *GVAX* GM-CSF-transfected pancreatic tumor vaccine, *ATRA* All-trans-retinoic-acid, *CAF* Cancer-associated fibroblast, *ECM* Extracellular matrix, *FAK* Focal adhesion kinase

## The path forward: precise medicine based on PDAC immunophenotyping

At present, our understanding of PDAC microenvironment is progressively deepening, and the immunophenotyping was designed more comprehensively on the basis of considering various components of TIME. However, a consensus regarding immunophenotyping for PDAC remains elusive. The current subtypes are still not sufficiently intricate, necessitating the determination of precise cells or signals that can distinguish between distinct subtypes. An innovative idea is that, for the existing genomics, proteomics, and other subtyping methods, we can further explore their immune characteristics [14, 204]. Whether the microenvironment is immune-activated or immune-deserted is determined by searching for the corresponding signature cells or markers. Thus, we establish a connection between diverse subtyping methodologies, serving as a cornerstone for advancing precision treatments. To enable precision medicine, it is crucial to design targeted drugs that align with characteristic cell types and signal markers associated with distinct subtypes. However, an important obstacle to achieving the goal of “more precise” is that there can be strong heterogeneity between different regions within the tumor, creating the possibility that subtyping of specimens derived from surgery or biopsy may not necessarily be representative of the entire tumor signature. Gratifyingly, a recent study isolated subTIMEs by deconvoluting the human pancreatic TIME through large-scale integration of regional multi-omics with clinical data and patient-derived preclinical models. The researchers identified three recurrent TIME phenotypes in PDAC: (a) “deserted” regions with thin, spindle-shaped fibroblasts and loose matured fibers, (b) “reactive” regions containing plump fibroblasts with enlarged nuclei, few acellular components, often rich in inflammatory infiltrate, and (c) regions with intermediate levels of these features. In the microenvironment, “reactive” subTIMEs rich in complex but functionally coordinated fibroblast communities were immune hot and inhabited by aggressive tumor cell phenotypes; the matrix-rich “deserted” subTIMEs harbored fewer activated CAFs and tumor-suppressive features yet were markedly chemoprotective and enriched upon chemotherapy [205]. In addition, subTIMEs differed strongly in composition: reactive/intermediate subTIMEs had increased staining for markers of T cells (CD3), macrophages (CD68), endothelial cells (CD31), and CAFs (αSMA), while deserted subTIMEs exhibited high collagen content and maturation and trended toward higher B cell marker (CD20) expression [205]. Collectively, this study revealed “hot” regions, “cold” regions and transition zones in PDAC. Regionally pinpointing the ecosystem status of a tumor might become routine testing for PDAC in the future because of its ability to guide more precise drug selection, delivery directions, and delivery modalities, literally treating every single patient as an exception.

In addition, an important factor affecting PDAC survival is the high incidence of metastasis, such as liver metastasis. Therefore, the evolutionary mechanism of the metastatic TIME also deserves special attention, as metastatic lesions may exhibit unique immune phenotypes and heterogeneity with the primary lesion. In a recent research, Zhang et al. performed single cell RNA sequencing for synchronously resected PDAC primary tumors and matched liver metastases. RGS5^+^ myCAFs, CCL18^+^ lipid-associated macrophages, S100A8^+^ TANs and FOXP3^+^ Tregs were more expressed in metastatic lesions [206]. In another study, the liver metastatic microenvironment was found to aggregate more M2 macrophages, which upregulate the expression of complement C1q and further promote PDAC cell migration [207]. In addition, it was found in a mouse PDAC model that compared to lung metastatic mice, the liver metastatic mice had more LAG3^+^ T cells, and CXCL12 signaling was upregulated, leading to stronger immune suppression [208]. This suggests the variability of immune regulatory pathways and immunophenotypes in different metastatic sites. Therefore, the treatment of PDAC not only considers the primary lesion, but also the metastatic lesion. We can incorporate the TIME features of metastatic PDAC into the corresponding subtypes. In conclusion, efforts aimed at understanding the pattern of tumor progression under distinct immunophenotypes and the development of tailored management approaches will undoubtedly drive the field of precision therapy for PDAC.

## Conclusions and perspectives

**Fig. 7:**
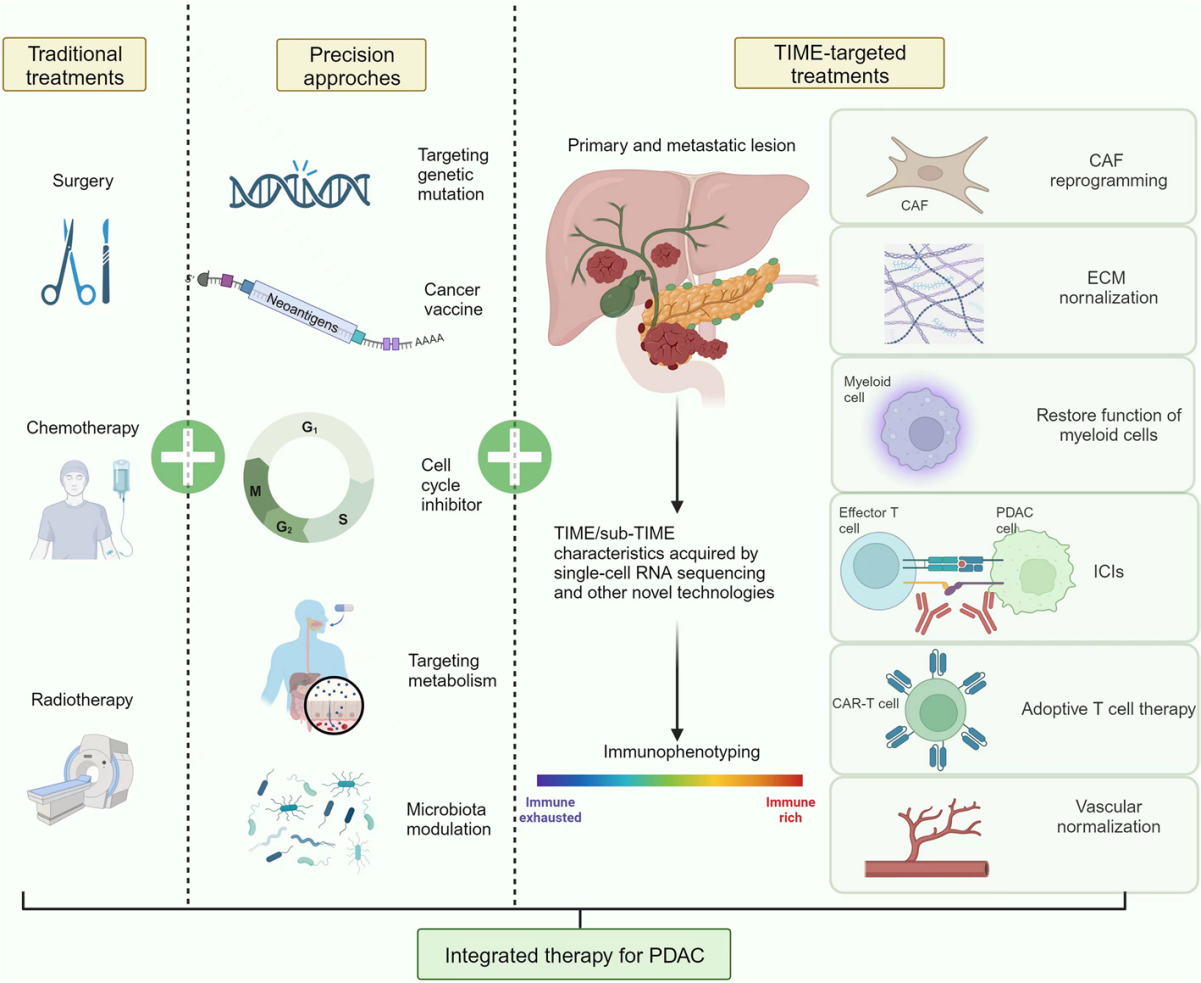
Future integrated therapeutic strategies for PDAC. The future goal of PDAC treatment is more precise. On the basis of traditional therapies such as surgery plus chemoradiotherapy, we design targeted drugs according to the changes of specific genes, the activation of tumor-promoting signaling pathways, the abnormal metabolic process, and the microbiota. Further, through in-depth exploration of the microenvironment of primary and metastatic lesions, we can find the commonness of the immune characteristics of a class of patients, and add drugs targeting TIME on the basis of immune subtypes, including CAF reprogramming, ECM normalization, restoration of myeloid cell function, ICIs, adoptive T cell therapy, and vascular normalization. This integrated strategy can meet the final requirement of precision treatment, that is, every patient should be treated as an exception

The PDAC microenvironment is characterized by its distinct features, including low immunogenicity, the abundant accumulation of CAFs and stromal components, a complex immunosuppressive signaling network, and the presence of exhausted effector T cells. These unique attributes set PDAC apart from other tumors and contribute to the challenges in achieving successful therapeutic outcomes. The pronounced heterogeneity of PDAC further limits the efficacy of treatment strategies. Consequently, a comprehensive understanding of PDAC immune subtypes becomes particularly essential. In recent years, substantial evidence related to the TIME has emerged, aided by advancements in mouse models, multi-omics technologies, high-throughput single-cell analysis, and cell profiling methods [130, 209–211]. These tools have deepened our insights into the intricate cellular and molecular complexities of PDAC and the dynamic interactions that fuel various immunosuppressive modes. Multiple innovative therapies targeting the TIME are currently in development. With this context in mind, a review of the latest advances in these areas is presented. Considering the characteristics PDAC TIME, the integrative therapy should be conducted on the basis of in-depth understanding of the subtypes. It includes: controlling the growth of the tumor itself, neutralizing immunosuppressive myeloid cells and stromal components, blocking immunosuppressive signals, conferring T cell antigen specificity, and enhancing the function of effector T cells. For certain targeted therapies of PDAC, such as those targeting metabolism, epigenetics, the tumor growth cycle and the microbiota, an innovative approach involves exploring the microenvironment and immune profiles of responsive patients [203, 212–216]. By doing so, we can tailor treatment selection and combine these therapies with ICIs for enhanced immunotherapeutic effects. We aim to propose an integrated therapy that can improve the clinical results of the existing PDAC, that is, the combination of surgery, chemotherapy, radiotherapy, targeted therapy, innate and adaptive immune regulation, matrix modulation, and microbiota-targeted therapies (Fig. 7). Furthermore, given the heterogeneity of PDAC, specific combinational treatment modalities for patients with commonalities in the TIME can be designed. Armed with a deep mechanistic understanding of the distinct immunosuppressive patterns present in different PDAC subtypes, we possess the potential to strategically guide precision medicine in the direction of TIME remodeling, ultimately aiming at PDAC eradication in the future.

## Data Availability

Data sharing not applicable to this article, as no datasets were generated or analyzed during the current study.

## Abbreviations

PDAC: Pancreatic ductal adenocarcinoma
OS: Overall survival
PFS: Progression-free survival
ICI: Immune checkpoint inhibitor
ORR: Objective response rate
MSI: Microsatellite instability
TMB: Tumor mutational burden
TIME: Tumor immune microenvironment
CTL: Cytotoxic T lymphocyte
TIL: Tumor infiltrating lymphocyte
TIGIT: T cell immunoreceptor with Ig and ITIM domains
PDCD1: Programed cell death 1
HAVCR2: Hepatitis A virus cellular receptor 2
LAG3: Lymphocyte-activation gene 3
LAYN: Layilin
ICOS: Inducible T-cell costimulatory
EOMES: Eomesodermin
GZMK: Granzyme K
RIPK2: Receptor-interacting protein kinase 2
DCR: Disease control rate
PARP: Polyadenosine-diphosphate-ribose polymerase
SBRT: Stereotactic body radiotherapy
CAR-T: Chimeric antigen receptor-T cell
PSCA: Prostate stem cell antigen
NAP: Neutrophil-activating protein
PSC: Pancreatic stellate cells
TCR: T-cell receptor
Treg: Regulatory T cell
C-FOXP3: Cancer Forkhead box protein 3
Breg: Regulatory B cell
STING: Stimulator of interferon gene
TAM: Tumor-associated macrophage
TAN: Tumor‐infiltrating neutrophil
DC: Dendritic cell
MDSC: Myeloid-derived suppressor cell
M-CSF: Macrophage colony-stimulating factor
OXPHOS: Oxidative phosphorylation
GARP: Glycoprotein A repetitions predominant
TLR: Toll-like receptor
Arg-1: Arginase-1
SIRPα: Signal-regulatory protein α
HDAC5: Histone deacetylase 5
BRD4: Bromodomain-containing protein 4
IRE: Irreversible electroporation
TNF: Tumor necrosis factor
Gas6: Growth arrest specific 6
HIF: Hypoxia-inducible factor
LAG3: Lymphocyte-activation gene 3
ROS: Reactive oxygen species
cDC1: Type I conventional dendritic cell
IRF8: Interferon regulatory factor-8
iNOS: Inducible nitric oxide synthase
GVAX: GM-CSF-transfected pancreatic tumor vaccine
CAF: Cancer-associated fibroblast
ECM: Extracellular matrix
NRP: Neural-like progenitor
iCAF: Inflammatory CAF
αSMA: α-Smooth muscle actin
myCAF: Myofibroblastic CAF
apCAF: Antigen-presenting CAF
FAP: Fibroblast activation protein
LRRC15: Leucine-rich repeat containing 15
csCAF: Complement-secreting CAF
meCAF: CAF with a highly activated metabolic state
EMT: Epithelial mesenchymal transition
SHH: Sonic hedgehog
CTGF: Connective tissue growth factor
LIF: Leukemia inhibitory factor
SPP1: Secreted phosphoprotein 1
Pin1: Peptidylpropyl isomerase
NetG1: Netrin G1
OSM: Oncostatin M
Col1: Collagens I
ATF4: Activating transcription factor 4
DDR1: Discoidin domain receptor 1
MRC1: Mannose receptor C-type 1
RNS: Reactive nitrogen species
NRF2: NF-E2-related factor 2
HA: Hyaluronan
FAK: Focal adhesion kinase
MMP: Matrix metalloproteinase
LPAR4: Lysophosphatidic acid receptor 4
ROCK: Rho-associated protein kinase
VEGF: Vascular endothelial growth factor
