# Correction: Tumor immune microenvironment-based therapies in pancreatic ductal adenocarcinoma: time to update the concept

**DOI:** 10.1186/s13046-024-02953-9

**Published:** 2024-01-18

**Authors:** Wenyu Luo, Ti Wen, Xiujuan Qu

**Affiliations:** 1 Department of Medical Oncology, The First Hospital of China Medical University, Shenyang, 110001 Liaoning China; 2 Key Laboratory of Anticancer Drugs and Biotherapy of Liaoning Province, The First Hospital of China Medical University, Shenyang, 110001 Liaoning China; 3 Clinical Cancer Research Center of Shenyang, the First Hospital of China Medical University, Shenyang, 110001 Liaoning China; 4 Key Laboratory of Precision Diagnosis and Treatment of Gastrointestinal Tumors, Ministry of Education, Shenyang, 110001 Liaoning China

**Correction:** ***J Exp Clin Cancer Res*** **43, 8 (2024)**

10.1186/s13046-023-02935-3

Following publication of the original article [1], authors noticed an error in the Funding section. The correct funding should read as:

- This work was supported by grants from Supporting the high-quality development of science and technology funding projects at China Medical University (To Xiujuan Qu, 2022JH2/20,200,039) (To Ti Wen, 2,023,020,792-JH2/202).

The correction does not affect the overall result or conclusion of the article. The original article has been corrected.
